# Genome-wide analysis of the human p53 transcriptional network unveils a lncRNA tumour suppressor signature

**DOI:** 10.1038/ncomms6812

**Published:** 2014-12-19

**Authors:** Yolanda Sánchez, Victor Segura, Oskar Marín-Béjar, Alejandro Athie, Francesco P. Marchese, Jovanna González, Luis Bujanda, Shuling Guo, Ander Matheu, Maite Huarte

**Affiliations:** 1 Center for Applied Medical Research, University of Navarra, 55 Pio XII Avenue, 31008 Pamplona, Spain; 2 Department of Gastroenterology, Donostia Hospital—Biodonostia Research Institute, University of Basque Country (UPV/EHU), Biomedical Research Center in the Network of Digestive and Hepatic Diseases (CIBERehd), Dr Begiristain Square, 20014 San Sebastian, Spain; 3 Department of Antisense Drug Discovery, Isis Pharmaceuticals, 2855 Gazelle Court, Carlsbad, California 92008, USA; 4 Neuro-Oncology Section, Oncology Department, Biodonostia Research Institute, Dr Begiristain Square, 20014 San Sebastian, Spain; 5 Ikerbasque, Basque Foundation for Science, Bilbao 48013, Spain

## Abstract

Despite the inarguable relevance of p53 in cancer, genome-wide studies relating endogenous p53 activity to the expression of lncRNAs in human cells are still missing. Here, by integrating RNA-seq with p53 ChIP-seq analyses of a human cancer cell line under DNA damage, we define a high-confidence set of 18 lncRNAs that are p53 transcriptional targets. We demonstrate that two of the p53-regulated lncRNAs are required for the efficient binding of p53 to some of its target genes, modulating the p53 transcriptional network and contributing to apoptosis induction by DNA damage. We also show that the expression of p53-lncRNAs is lowered in colorectal cancer samples, constituting a tumour suppressor signature with high diagnostic power. Thus, p53-regulated lncRNAs establish a positive regulatory feedback loop that enhances p53 tumour suppressor activity. Furthermore, the signature defined by p53-regulated lncRNAs supports their potential use in the clinic as biomarkers and therapeutic targets.

## Abstract (web summary)

Several studies have shown that p53 regulates the expression of some long noncoding RNAs (lncRNAs) implicated in apoptosis and proliferation. Here, by integrating RNA-seq and ChIP-seq analyses, the authors identify p53-regulated lncRNAs in the HCT116 colorectal cancer cell line.

## Graphical abstract

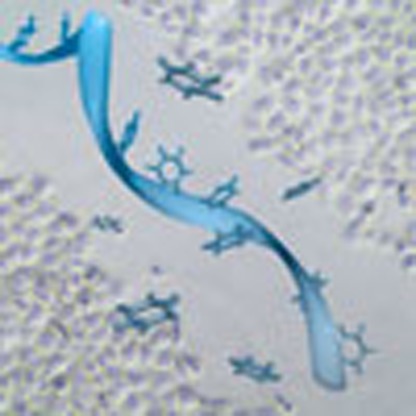

The transcription factor p53 is the most prominent human tumour suppressor. p53 is essential for the cellular response to DNA-damaging stimuli to maintain genomic integrity of cells, mainly by activating a gene expression programme that leads to cell cycle arrest or elimination of the damaged cells through programmed cell death. The vast majority of the p53 downstream effects are mediated through its intrinsic nature as transcription factor^1^. On cellular stress, p53 protein is stabilized and can recognize its target genes through binding to a consensus response element (p53RE) located proximal to the transcription start site (TSS) at the gene promoter, the first intron or even further downstream of the gene^2^. For decades, researchers have focused their attention on the protein-coding genes regulated by p53, which led to the discovery of a large set of proteins involved in the p53 response. However, recent progress has suggested that a significant number of p53REs lie on noncoding regions of the genome and that some of these genomic loci are transcribed into long noncoding RNAs (lncRNAs).

LncRNAs are transcripts longer than 200 nucleotides that lack functional open reading frames^3^,^4^. Similarly to mRNAs, lncRNAs are frequently polyadenylated and spliced, and their promoters are subjected to regulation by transcription factors such as p53. Distinctive features of lncRNAs are their highly specific expression patterns and relatively low conservation across species, consistent with their role as regulatory molecules that fine-tune gene expression^4^,^5^,^6^,^7^.

Few lncRNAs have been studied in some depth. These show the important roles of lncRNAs in many processes that involve gene regulation, such as cellular differentiation, proliferation, dosage compensation and chromosomal imprinting^3^,^8^,^9^,^10^,^11^,^12^. Given their physiological activities, the deregulation of lncRNAs is one of the underlying causes of human disease, including cancer, and they emerge as promising targets for novel therapies^13^,^14^,^15^.

Our work and others’ have shown that p53 regulates the expression of some lncRNAs. For instance, we identified *lincRNA-p21* (ref. 16) and *Pint*^17^, which modulate cellular apoptosis and proliferation in mouse cells. Studies by other groups lead to the identification of *PANDA*^18^, a lncRNA able to inhibit cellular apoptosis in human fibroblasts, *lincRNA-RoR* and *loc285194* (refs 19, 20), reported as post-tanscriptional regulators in the p53 pathway, and *TUG-1*, which controls proliferation in human non-small cell lung cancer^4^,^21^.

Although these studies underscore a functional role of lncRNAs in the p53 pathway, the extent of the contribution of lncRNAs to the p53 response to DNA damage in human cells still remains poorly understood.

In this study, we integrate genome-wide expression data obtained by RNA sequencing (RNA-seq) with p53 ChIP-seq data of human cancer cells treated or not with a DNA-damaging drug. The combination of these experimental approaches allowed us to relate the active binding of p53 to the expression of protein-coding and -noncoding regions of the genome, including regions poorly or not annotated previously. We also provide experimental evidence of the contribution of a subset of lncRNAs to the p53 human transcriptional network and biological activity. Finally, we show a tumour suppressor signature defined by p53-regulated lncRNAs.

## Results

### Hundreds of lncRNAs are affected by DNA-damage treatment in human cells

**Figure 1:**
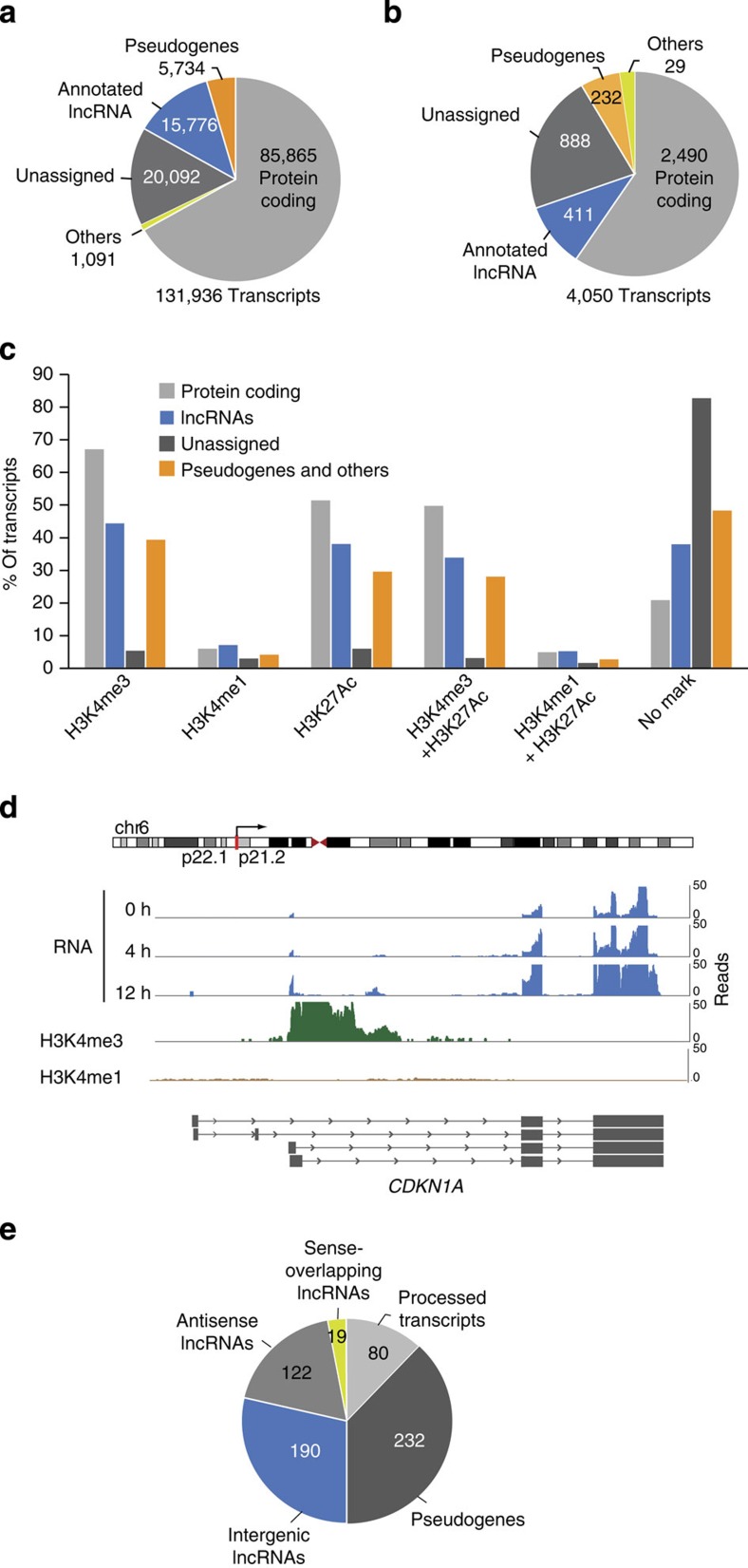
Genome-wide analysis of the DNA-damage transcriptome of HCT116 cells. (**a**) Biotype distribution of all the transcripts identified by RNA-seq analysis generated from HCT116 cells untreated or treated with DNA damage annotated relatively to ENCODE v19. (**b**) Biotype distribution of the differentially expressed transcripts following DNA-damage treatment with 5-FU for 12 h. (**c**) Percentage of the transcripts identified by RNA-seq with H3K4me3, H3K4me1 or/and H3K27ac marks at the 5′ position of the defined transcript structures. (**d**) Schematic representation of the chromosomal location of the *CDKN1A* gene locus, RNA expression detected by RNA-seq of DNA-damage-treated cells and, H3K4me3 and H3K4me1 levels of HCT116 untreated cells and *CDKN1A* transcript isoforms as assembled by Cufflinks. (**e**) Subtypes’ distribution of the lncRNAs found differentially expressed following 5-FU treatment.

We set out to investigate the polyadenylated human transcriptome regulated by p53. To that end, we used as a model the HCT116 colorectal cancer cell line, which has been previously reported to have an intact p53 response^22^ (Supplementary Fig. 1a). To induce p53 protein stabilization and transcription of its target genes, cells were treated with the DNA-damage-inducing drug 5-fluorouracil (5-FU) for different times (0, 4 and 12 h). We then isolated the polyadenylated RNA fraction and performed strand-specific paired-end RNA Illumina sequencing with reads of 150 base pairs (bp) length (Supplementary Fig. 1b). On average, we obtained 170M mapped reads per experimental condition, which were assembled using Cufflinks and Cuffmerge^23^ (See Methods and Suplementary Methods). In total, 131,936 transcripts were successfully assembled, of which 85% were annotated according to Gencode v19 (refs 24, 25). Out of the annotated transcripts, 76% (85,865) were identified as protein-coding mRNAs, while the remaining 24% were classified as different types of noncoding transcripts. A total of 14% (15,776) were defined as lncRNAs, including antisense, intergenic, processed transcripts, sense-overlapping and sense-intronic lncRNAs, while the remaining 10% (6,825) of annotated transcripts corresponded to other types of noncoding RNAs, such as transcripts derived from pseudogenes, retained introns and pri-microRNAs (Fig. 1a). This relative distribution of transcripts is similar to that described by Encode^25^. We also found a large number of unassigned transcripts (20,092), many of which were unspliced and which could partially be an artefact caused by incomplete determination of the transcript structures. To address this point, we analysed publicly available ChIP-seq data from HCT116 cells^26^. While the genomic region ranging from −5kb to +5kb around the 5′ end of 83% of the unassigned transcripts defined by our RNA-seq was not associated with an active transcription chromatin mark (that is, H3K4me3, H3K4me1 or H3K27Ac; Fig. 1c), the remaining 17% were enriched by at least one of these histone marks at their 5′ end, suggesting the presence of a promoter driving their expression.

Next, to determine what transcripts are perturbed by the DNA-damage treatment, we applied Cuffdiff 2 (ref. 27; see Methods). Comparing HCT116 cells treated with DNA damage for 12 h to untreated cells, 4,050 transcripts were found differentially expressed (*P*<0.01, Cuffdiff differential expression analysis based on the beta-negative binomial distribution) in at least one of the two replicates. A total of 1,738 of these RNAs were upregulated, while 2,313 were downregulated upon drug treatment. Out of the total number of transcripts differentially expressed, 60% corresponded to protein-coding genes (Fig. 1b; Supplementary Table 1) and, as expected, included well-known canonical p53 target genes such as *CDKN1A*, *BBC3*, *PCNA* and *BAX* (Fig. 1d; Supplementary Table 1). To obtain a global view of the efficacy of the DNA-damage treatment as well as the activation of the p53 response, the whole set of protein-coding genes differentially expressed in our RNA-seq was used to predict upstream regulators and cellular pathways *in silico*. As expected, the most significant upstream regulator predicted was the transcription factor p53 (*P*=6.04E−37, Ingenuity Fisher’s exact test; Supplementary Fig. 1c) and the most enriched canonical pathway was the p53 signalling, followed by ataxia-telangiectasia mutated (ATM) signalling pathway (Supplementary Fig. 1d). These results confirmed that the genes selected by our analysis are enriched in those of the p53 transcriptional response to DNA damage.

Besides the protein-coding transcripts, we aimed to identify lncRNAs differentially expressed following DNA damage. A total of 1,531 RNAs with expression values significantly affected by the DNA-damage treatment were found (Supplementary Table 1). A total of 633 corresponded to lncRNAs, distributed as intergenic (190), antisense (122), sense-overlapping lncRNAs (19), processed transcripts (80) and transcripts derived from pseudogenes (232; Fig. 1e). In addition, we found 888 unassigned transcripts affected by the drug treatment that, as discussed above, may account in part for artefacts due to technical limitations associated with RNA-seq data production and analysis.

To validate our results, we performed quantitative reverse transcription PCR (qRT–PCR) for a selection of 26 differentially expressed lncRNAs on HCT116 cells treated with DNA damage (23 upregulated and 3 downregulated on DNA damage; Supplementary Fig. 2) and confirmed the differential expression for 23 (20 up- and 3 downregulated) out of the 26 lncRNAs (88%).

In summary, we compiled a genome-wide catalogue of protein-coding and noncoding transcripts affected or not by DNA damage in HCT116 cells and identified a set of lncRNAs that are differentially expressed upon DNA damage in human cells.

### p53 associates to multiple lncRNA gene loci

Our gene expression analysis identified transcripts with altered expression upon DNA damage, which comprises genes of the p53 response. However, it was unable to distinguish those directly regulated by p53.

**Figure 2:**
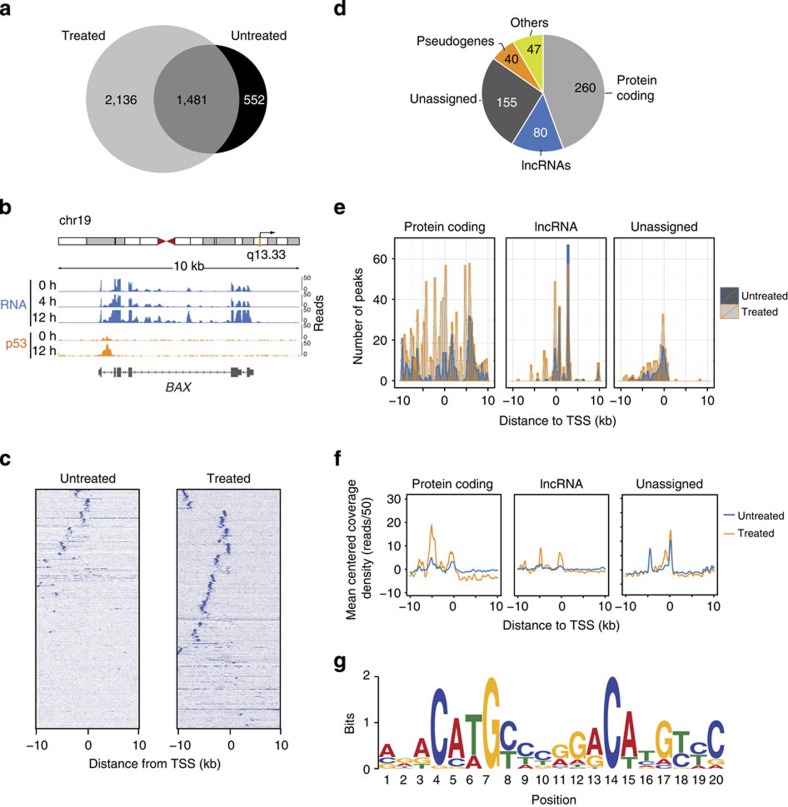
Genome-wide analysis of p53 binding. (**a**) Total number of p53-binding loci identified by p53 ChIP-seq analysis of HCT116 cells untreated or treated with DNA damage for 12 h. (**b**) Schematic representation of the chromosomal location of the *BAX* gene locus, RNA-seq and p53 ChIP-seq signals at 0 and 12 h of 5-FU treatment. (**c**) Density heatmap of the p53-binding sites across 10 kb from the TSS of genes in untreated and treated HCT116 cells. (**d**) Distribution by biotypes of genes differentially expressed and bound by p53 upon DNA-damage treatment for 12 h. (**e**) Number of p53-binding sites and position along 10 kb from the TSS of the nearest gene separated into protein-coding, lncRNA or unassigned gene loci. (**f**) Distribution of ChIP-seq reads along 10 kb of distance from the nearest TSS of the indicated gene types. (**g**) p53 consensus motif defined by MEME motif analysis of the sequences of p53 ChIP-seq peaks of genes upregulated by DNA damage (*P*=4.9E−22, TOMTOM match statistic).

To discern p53 direct targets, we performed p53 ChIP-Seq on untreated HCT116 cells as well as cells treated with DNA damage (12 h of 5-FU treatment). ChIP-seq analysis identified a total of 3,617 p53 peaks in the DNA-damage-treated cells (Fig. 2a). Interestingly, p53 was bound to 1,481 sites even in the absence of DNA damage, indicating the p53 basal activity in the cells. In many cases, the peaks detected even in the absence of treatment corresponded to known bona fide p53 target genes such as *CDKN1A*, *BAX* and *BBC3* (Supplementary Table 2). However, the intensity of p53 binding at these loci, as well as the number of peaks and average global signal intensity, was increased upon DNA-damage treatment (Fig. 2a–c,e; Supplementary Fig. 3a–d).

The p53 ChIP-seq peaks were annotated relative to Gencode v19 and to our RNA-seq analysis in the case of the novel transcripts not previously annotated. A total of 582 gene loci were bound by p53, of which 260 are protein-coding genes, 80 lncRNAs and 155 unassigned genomic regions (Fig. 2d). Analysis of the relative position of the p53-binding peaks showed that 60% of them localized within 10 kb from the TSS of the nearest gene (proximal peaks), while the remaining 40% lied more than 10 kb apart (distal peaks). Interestingly, in the case of protein-coding genes, the positional distribution of the p53 proximal peaks was relatively uniform within the 20 kb window (Fig. 2e), while the majority of the proximal p53 peaks associated with lncRNAs were concentrated less than 5 kb downstream of the TSS (Fig. 2e). On the other hand, the analysis of the signal distribution showed that the ChIP-seq signal was enriched around the TSS of genes and also at 5 kb upstream of the TSS (Fig. 2f). This pattern of distribution of p53 signal was common to all gene types, independently of their coding or noncoding nature, indicating that the most intense p53 binding occurs around the 0 and −5 kb positions.

### p53 regulates the expression of lncRNAs upon DNA damage

Next, to determine the effect of p53 binding on gene expression, we compared the p53 ChIP-seq data with the RNA-seq analysis. In DNA-damage-treated cells, p53 was found bound to 109 genes that also showed at least one transcript differentially expressed when only considering the p53 proximal peaks (<10 kb of distance to TSS; Fig. 2e). A total of 75% of these p53 directly regulated genes were upregulated on DNA damage, while only 25% were downregulated (Supplementary Table 3). To confirm the presence of the p53-binding motifs, we performed *de novo* motif analysis using the genomic sequences associated with the p53-binding peaks of those transcripts differentially expressed (see Methods). In agreement with the binding of p53, the p53 consensus motif was found highly enriched across the p53-bound loci (motif similarity *P*=2.12E−10, TOMTOM match statistic; Fig. 2g), confirming the presence of p53REs in these genomic regions.

We focused our attention on the noncoding transcripts differentially expressed on DNA damage and directly regulated by p53, which we named p53-regulated lncRNAs (*PR-lncRNAs*). We identified a total of 27 transcripts (from 25 genomic loci; Supplementary Table 4). Eleven of them were annotated by Gencode v19 as intergenic lncRNAs (5), antisense lncRNAs (4) or processed transcripts (2). In addition, we found 16 unassigned transcripts, which in some cases had been annotated in previous studies as lncRNAs (Supplementary Table 4). By analysis of enrichment of chromatin marks, relative position to protein-coding genes and quantification of their coding potential (CPAT score^28^), we concluded that, while 7 of the 16 unassigned transcripts could be an artefact of the RNA-seq assembly (for example, extended 3′ untranslated region of the nearest gene), the remaining 9 likely represent p53-regulated lncRNAs.

**Figure 3:**
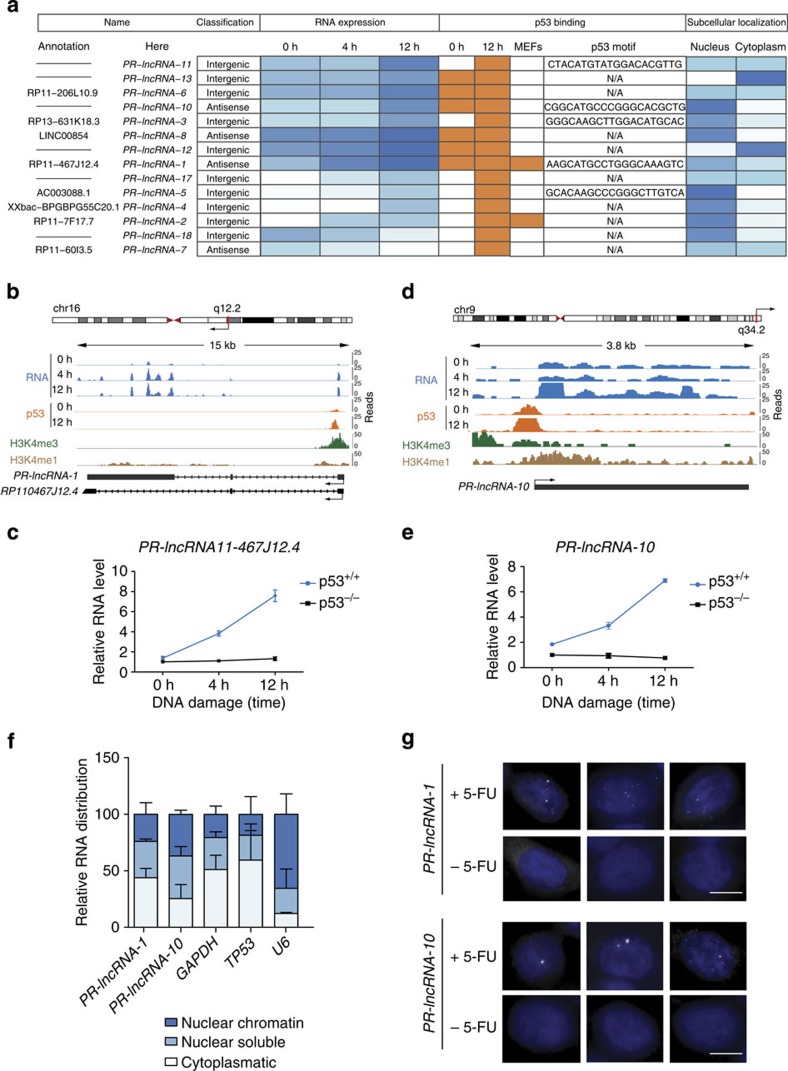
Human lncRNAs directly regulated by p53. (**a**) Summary table of lncRNAs directly regulated by p53. Different shades of blue indicate the RNA expression values for each lncRNA under the different conditions of treatment. The presence of p53 binding to the lncRNA loci in HCT116 cells at 0 and 12 h of 5-FU treatment or in MEFs treated with doxorubicin is indicated in orange, and the p53-binding sequence motif obtained by MEME analysis is shown. The relative subcellular localization of the lncRNAs determined by subcellular fractionation followed by qRT–PCR is indicated with colours from white to dark blue according to increasing RNA levels. Values are the average of three biological replicates. (**b**–**d**) Schematic representation of the chromosomal locations of the *PR-lncRNA-1* and *PR-lncRNA-10* gene loci. RNA expression (RNA-seq signal), p53, H3K4me3 and H3K4me1 ChIP-seq peaks and transcript structures as assembled by Cufflinks (grey) or annotated by Gencode (black). Validation by qRT–PCR of the *PR-lncRNA-1* (**c**) and *PR-lncRNA-10* (**e**) in an independent experiment with HCT116 p53^+/+^ and p53^−/−^ untreated or treated with DNA damage for 4 and 12 h. Values represent the mean±s.d. of three biological replicates. (**f**) Subcellular localization. Percentage of total RNA found in the nuclear fraction bound to chromatin, nuclear soluble and cytoplasmic fractions in the HCT116 p53^+/+^ cells determined by qRT–PCR. Values represent the mean±s.d. of three biological replicates. (**g**) RNA fluorescence *in situ* hybridization of *PR-lncRNA-1* and *PR-lncRNA-10* in HCT116 p53^+/+^ cells untreated (−5-FU) or treated (+5-FU) with 5-FU for 12 h. White line indicates 10 μm.

These results were independently validated by qRT–PCR, observing that 6 out of the 9 unassigned transcripts and the 11 annotated lncRNAs presented the expected expression pattern in p53 WT cells. Furthermore, by analysing their expression in the isogenic HT116 p53^−/−^ cell line^22^, we confirmed that their differential expression is dependent on the presence of p53 in the cells (Supplementary Fig. 2). A summary table of the p53-regulated lncRNAs (excluding the processed transcripts) and some of their features is presented in Fig. 3a.

To further characterize the p53-regulated lncRNAs, and given the prominent use of mouse models for p53 studies, we wanted to determine the extent of conservation of p53 regulation in mouse cells. We therefore compared our human p53 ChIP-seq data with previously published p53 ChIP-seq data from mouse embryonic fibroblasts (MEFs) treated with the DNA-damage drug doxorubicin^29^. The genome-wide analysis revealed that 25% of the p53-binding loci found in the HCT116 human cancer cells were also recognized by p53 in MEFs (Supplementary Table 5). Interestingly, when we quantified only the peaks associated with the differentially expressed genes, the proportion increased to 50%, suggesting that p53 activity on DNA-damage treatment is highly (but not totally) conserved even between different cell types of distant mammalian species.

Interestingly, the transcripts with conserved regulation between mouse and human cells included three noncoding RNAs (two lncRNAs and one processed transcript; Supplementary Table 5; Fig. 3a). To confirm this observation, we performed qRT–PCR in p53^+/+^ and p53^−/−^ MEFs treated with doxorubicin on the mouse orthologous regions corresponding to the noncoding RNAs. For all of them, we confirmed higher RNA expression in the presence of p53 and increased expression upon DNA-damage treatment (Supplementary Fig. 3g).

In summary, we have identified a high-confidence set of 18 lncRNAs that are directly regulated by p53 upon DNA-damage treatment in human cells, 3 of which show conserved regulation in mouse cells.

### p53-regulated lncRNAs contribute to the p53 transcriptional network

After identifying human lncRNAs that are bona fide p53 transcriptional targets, we wanted to determine to what extent they contribute to the gene expression changes caused by p53 upon DNA damage. We selected two representative lncRNAs: (i) *PR-lncRNA-1*, a 5,250-nt-long multiexonic intergenic transcript, annotated by Gencode as RP11-467J12.4, predominantly localized in the nucleus of cells and shown to be p53 regulated in both human and mouse cells (Fig. 3a–c,f,g) and (ii) *PR-lncRNA-10*, a non-annotated monoexonic intergenic lncRNA of 2,780 nt of length, localized in the nucleus and with no detected p53 regulation in mouse (Fig. 3a,d–g).

**Figure 4:**
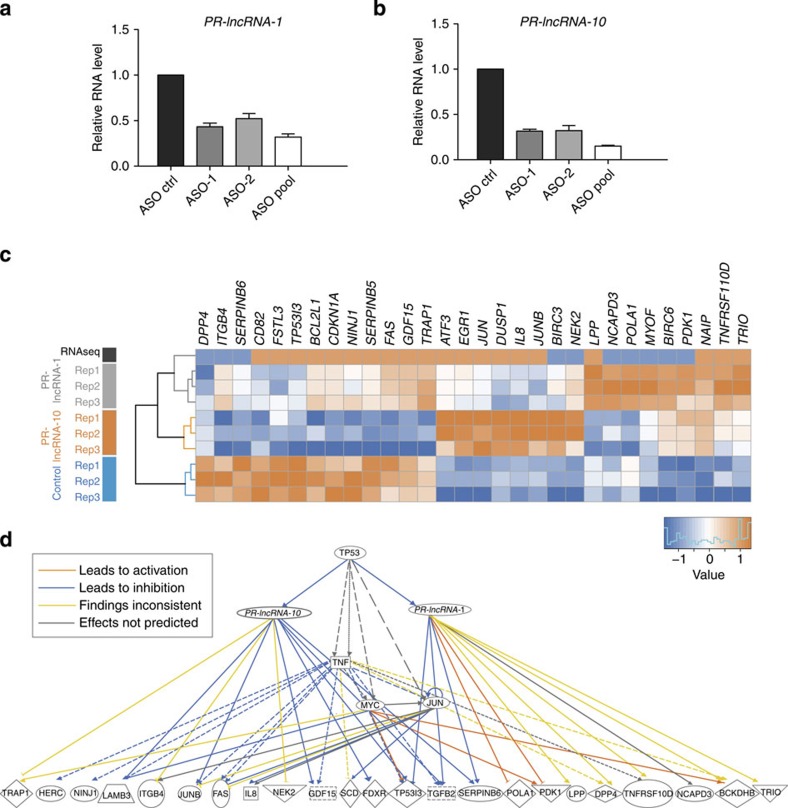
Role of the p53 targets *PR-lncRNA-1* and *PR-lncRNA-10* in the p53 transcriptional response. (**a**,**b**) HCT116 cells were transfected with a non-targeting ASO (ASO Ctrl) or with two different ASOs (ASO-1 and ASO-2) targeting *PR-lncRNA-1* (**a**) or *PR-lncRNA-10* (**b**), separately or in combination (ASO pool). *PR-lncRNA-1* and *PR-lncRNA-10* RNA knockdown efficiencies were determined by qRT–PCR. Graphs represent the mean (±s.d.) of three independent experiments. (**c**) Expression levels of representative genes found differentially expressed in at least one of the microarray analysis (*PR-lncRNA-1* or *PR-lncRNA-10* depletion) and in HCT116 when treated with 5-FU (RNA-seq analysis). Colours from dark blue to orange indicate increasing RNA levels in each of the experiments. (**d**) Network connecting p53, *PR-lncRNA-1* and *PR-lncRNA-10* with some of the commonly regulated genes involved in the DNA-damage response as predicted by Ingenuity Pathway Analysis.

To deplete cells of these lncRNAs, we designed several antisense oligonucleotides (ASOs) to achieve specific degradation and selected the two most effective ASOs in reducing the lncRNA levels (Fig. 4a,b). For each lncRNA, we then transfected HCT116 cells with two targeting ASOs, as well as a non-targeting ASO as control for 36 h and then treated the cells with 5-FU for 12 h to induce p53. Total RNA was isolated and gene expression analysis was performed by microarray (See Methods and Suplementary Methods).

The analysis revealed that the depletion of *PR-lncRNA-1* affected the expression of 932 genes (748 up- and 184 downregulated), while *PR-lncRNA-10* reduction caused the altered expression of 1,647 genes (1,290 up- and 357 downregulated; *B*>0, limma B-statistics or log odds; Supplementary Table 6). Interestingly, the relationship between p53 and these two lncRNAs was confirmed by the prediction of p53 as a significant upstream regulator for both sets of genes (*P*=2.12E−05 for *PR-lncRNA-1* and *P*=1.00E−12 for *PR-lncRNA-10*, Ingenuity Fisher’s exact test). The gene expression changes observed by microarray analysis were validated in independent experiments where we transfected separately the individual ASOs to knockdown the lncRNAs and performed qRT–PCR for a panel of representative genes. We confirmed the expression changes for 12 out of 17 (*PR-lncRNA-1*) and 16 out of 18 (*PR-lncRNA-10*) differentially expressed genes. Few mRNAs that did not show a statistically significant differential expression by qRT–PCR, still showed the same trend (up- or downregulated) observed in the microarray analysis (Supplementary Figs 4a and 5a). In addition, we performed independent validations by transfecting the ASOs into HCT116 p53^−/−^ cells with or without DNA-damage treatment. Under these conditions, we could not detect any effect of the ASO treatment on the validation gene set, confirming that the effects observed are specific of the p53-dependent expression of *PR-lncRNA-1* and *PR-lncRNA-10* (Supplementary Figs 4b,c and 5b,c).

We next compared the effect of *PR-lncRNA-1* and *PR-lncRNA-10* depletion with the observed gene expression changes caused by DNA damage in HCT116 cells. Out of the 69 protein-coding genes regulated by *PR-lncRNA-1* that consistently changed with DNA-damage treatment, many were related to induction of apoptosis and proliferation. For instance, we found the apoptosis regulators *BCL2L* and BIRC3, the DNA polymerase subunit POLA1 or the growth factor TGFB2 (Fig. 4c,d; Supplementary Table 7). On the other hand, 109 of the protein-coding genes regulated by *PR-lncRNA-10* constituted another component of the DNA-damage response, and included the cell cycle inhibitor CDKN1A, the transcription factor JUNB or the apoptosis regulators BIRC6, TP53I3 and FAS among many others (Fig. 4c,d; Supplementary Table 7). In agreement with a potential role of these lncRNAs in the p53 response to DNA damage, p53 was found to be the most significant predicted upstream regulator and enriched pathway for the sets of genes affected by both DNA damage and *PR-lncRNA-1* or *PR-lncRNA-10* (*P*=1.0E−07 or *P*=2.1E−03, respectively). This is illustrated by the network depicted in Fig. 4d, which represents the predicted relationships between some of the genes co-regulated by p53 and *PR-lncRNA-1* or *PR-lncRNA-10*.

Altogether, the results shown here suggest that *PR-lncRNA-1* and *PR-lncRNA-10* are active components of the p53 transcriptional response and that they modulate the gene expression response to DNA damage downstream of p53.

### *PR-lncRNAs* required for efficient binding of p53 to gene targets

Our results showed that *PR-lncRNA-1* and *PR-lncRNA-10* are not just directly regulated by p53, but are involved in the regulation of genes of the p53 pathway. We then investigated how these lncRNAs could affect the expression of their targets. While the microarray analysis showed changes in the steady-state levels of the mRNAs regulated by *PR-lncRNA-1* and *PR-lncRNA-10*, it did not distinguish whether the changes are taking place at the transcriptional or post-transcriptional level. To address this point, we analysed the stability of *PR-lncRNA-1* and *PR-lncRNA-10* target mRNAs upon knockdown of the lncRNAs by blocking their transcription with actinomycin-D treatment. These experiments did not show significant changes in the stability of the mRNAs analysed, suggesting that *PR-lncRNA-1* and *PR-lncRNA-10* do not act post-transcriptionally on these mRNAs, but rather regulate their expression at the transcriptional level (Supplementary Fig. 6).

**Figure 5:**
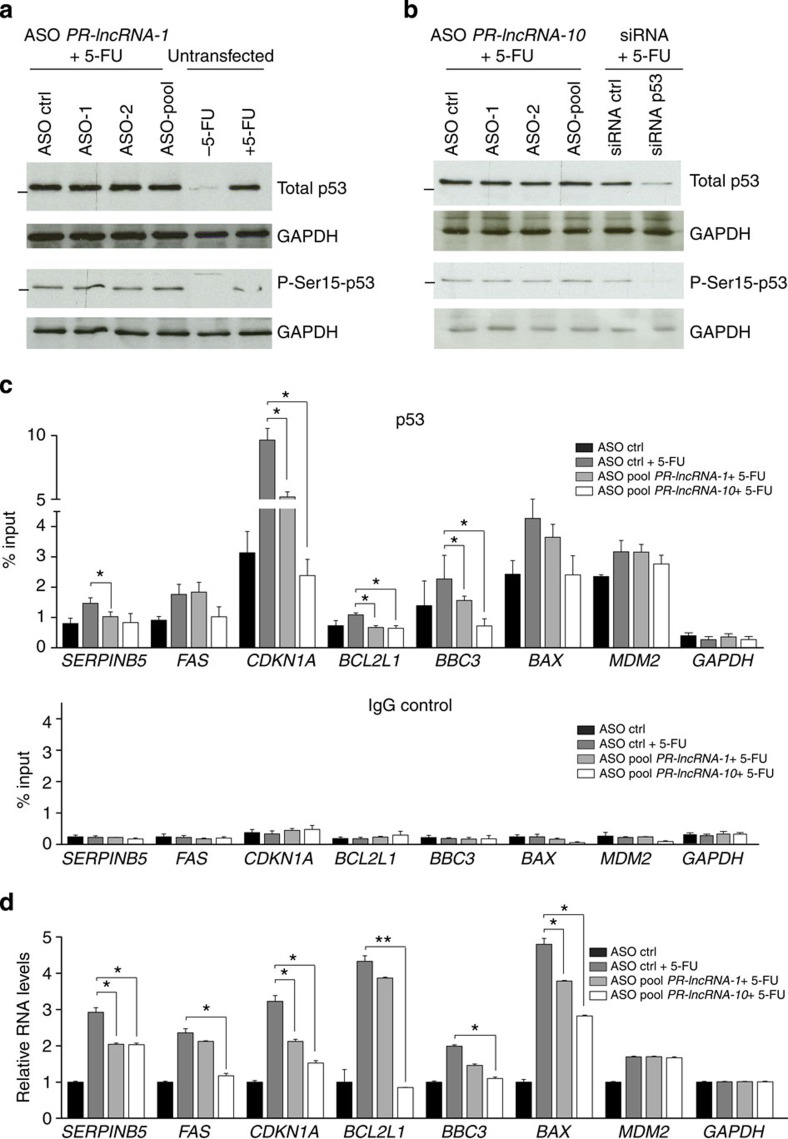
*PR-lncRNA-1* and *PR-lncRNA-10* are required for the transcriptional activation of some genes by p53. (**a**,**b**) Western blot analysis of total p53 and phospho-p53 (Serine 15) on HCT116 cells transfected with ASOs for *PR-lncRNA-1* (**a**) or *PR-lncRNA-10* depletion (**b**) and treated as indicated. GAPDH is shown as a loading control. MW mark indicates 50 kDa. (**c**) p53 ChIP of p53 direct target genes regulated by *PR-lncRNA-1* and *PR-lncRNA-10*. ChIP enrichment of p53 and control IgG of the indicated loci in HCT116 cells after the transfection with ASO pool for *PR-lncRNA-1*, *PR-lncRNA-10* or ASO control for 36 h and treatment with 5-FU for 12 h. *GAPDH* promoter was included as a negative control. The mean±s.d. of three biological replicates, and the significant differences relative to the condition ASO ctrl +5-FU are shown. (**d**) Relative expression levels of the p53 direct target genes regulated by *PR-lncRNA-1* and *PR-lncRNA-10* in HCT116 cells treated like in **c** and determined by qRT–PCR. Values are the average of three biological replicates, and the significant differences relative to the condition ASO ctrl +5-FU are shown. All graphs (**c**,**d**) represent the mean (±s.d.) of at least three independent experiments. Significance was determined by two-tailed unpaired *t*-test. *P<0.05 and **P<0.01.

As discussed above, the transcription factor p53 is predicted to be the upstream regulator of the genes affected by *PR-lncRNA-1* and *PR-lncRNA-10* knockdowns. We therefore hypothesized that *PR-lncRNA-1* and *PR-lncRNA-10* could affect p53 activity. We excluded the possibility that p53 gene expression was affected, as the microarray analyses and additional qRT–PCR validations clearly showed that p53 mRNA levels were not changed on *PR-lncRNA-1* nor *PR-lncRNA-10* knockdown (Supplementary Figs 4 and 5). As p53 is tightly regulated at the protein level, we analysed total p53 protein level in *PR-lncRNA-1* and *PR-lncRNA-10* knockdown conditions. However, we did not detect any differences upon knockdown of the lncRNAs (Fig. 5a,b). Similarly, we did not detect any changes in the levels of phosphorylated p53 protein at serine 15, which is generally thought to be involved in the activation of p53 after DNA damage^30^. We concluded that neither *PR-lncRNA-1* nor *PR-lncRNA-10* affects p53 protein levels or phosphorylation.

We then tested whether the ability of p53 to transcriptionally activate some target genes could be influenced by *PR-lncRNA-1* or *PR-lncRNA-10.* To that end, we selected a set of genes that are p53 direct transcriptional targets, that is, are directly bound by p53 and change their expression on DNA damage (*SERPINB5*, *FAS*, *CDKN1A*, *BCL2L1*, *BBC3*, *BAX* and *MDM2*; see Supplementary Table 3). Most of the selected genes (*SERPINB5*, *FAS*, *CDKN1A* and *BCL2L1*) were also found altered in our microarray analysis by the knockdown of *PR-lncRNA-1* and/or PR-*lncRNA-10* (Supplementary Table 8; Fig. 4c,d). We then performed p53 ChIP in the presence and absence of *PR-lncRNA-1* or *PR-lncRNA-10* depletion. On *PR-lncRNA-1* and/or *PR-lncRNA-10* depletion, we observed a significant decrease in the binding of p53 to the p53REs of *SERPINB5*, *CDKN1A*, *BCL2L1* and *BBC3* genes, although the decrease was generally more pronounced on *PR-lncRNA-10* inhibition (Fig. 5c). Furthermore, when the mRNA levels were quantified in the same experiments, we observed a decrease in the expression of the genes in correlation with the decrease in p53 binding to their promoters upon the lncRNAs depletion (Fig. 5d).

Taken together, these results suggest that p53 requires *PR-lncRNA-10* and, in lesser extent, *PR-lncRNA-1* to efficiently bind and activate some of its direct transcriptional targets.

### *PR-lncRNA-1* and *PR-lncRNA-10* are negative regulators of cell survival and proliferation

**Figure 6:**
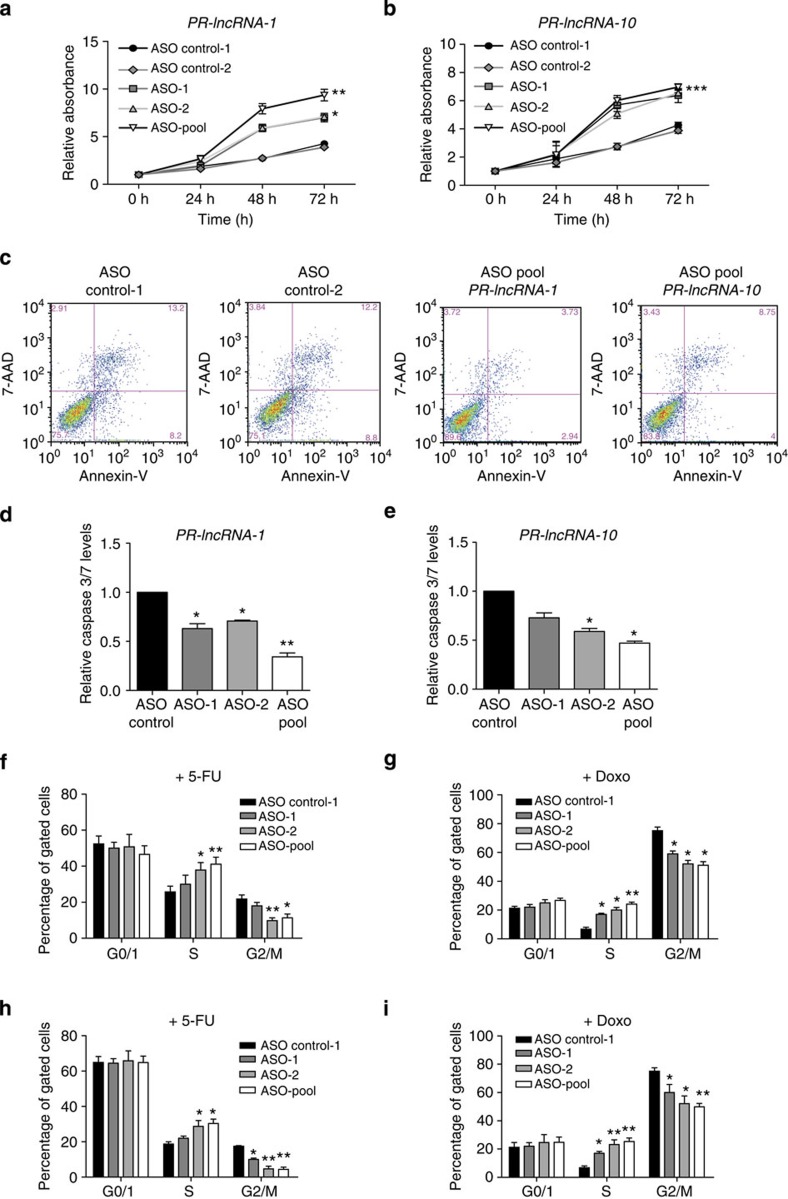
*PR-lncRNA-1* and *PR-lncRNA-10* modulate cell proliferation and apoptosis. (**a**,**b**) HCT116 cells were transfected with two separate non-targeting ASOs (Ctrl-1 and Ctrl-2) or with two different ASOs (ASO-1 and ASO-2) targeting *PR-lncRNA-1* or *PR-lncRNA-10*, separately or in combination (ASO pool), and treated with 150 nM DNA-damage Doxo for 12 h. Cell proliferation was measured by MTS assay up to 72 h (**a**,**b**). (**c**) Percentage of apoptotic cells measured by annexinV-7AAD (7-aminoactinomycin D) staining on cells transfected with the indicated ASOs and treated with the 350 μM DNA-damage drug 5-FU for 12 h. (**d**,**e**). Apoptosis was determined by quantification of caspase 3/7 levels in cells treated with 5-FU for 12 h and transfected with the indicated ASOs. (**f**–**i**) Cell cycle phase distribution analysed by propidium iodide staining of cells transfected with ASOs specific for *PR-lncRNA-1* (**f**,**g**) or *PR-lncRNA-10* (**h**,**i**) depletion and treated with the indicated drugs for 12 h. All graphs (**a**–**i**) represent the mean (±s.d.) of at least three independent experiments. Significance was determined by two-tailed unpaired *t*-test. *P<0.05 and **P<0.01.

The gene expression analysis revealed that *PR-lncRNA-1* and *PR-lncRNA-10* modulate the expression of several genes related to cell cycle control and apoptosis induction, which are the major functional outcomes of p53 activation. To determine the role of the p53-regulated lncRNAs in this context, we monitored cell proliferation after depletion of *PR-lncRNA-1* or *PR-lncRNA-10* in the presence or absence of DNA-damage induction. HCT116 cells were treated with doxorubicin (doxo) to induce DNA damage, as this drug induces expression of p53 and its target genes, including *PR-lncRNA-1* and *PR-lncRNA-10* (Supplementary Fig. 7a,b), but is not as strong apoptosis inducer as 5-FU, allowing us to carry out experiments for several days. Cell proliferation assays were performed with HCT116 cells showing a significant increase in the number of viable cells when depleted of *PR-lncRNA-1* or *PR-lncRNA-10* compared with the controls (Supplementary Fig. 7c,d), both in the presence and absence of drug treatment, although the difference in proliferation between lncRNA-depleted cells and controls was more marked in cells treated with DNA damage (Fig. 6a,b). These results suggested that both *PR-lncRNA-1* and *PR-lncRNA-10* may contribute to the p53 pro-apoptotic and/or cell cycle regulatory functions.

The major effect of DNA damage on HCT116 cells is a generalized cellular apoptosis, which reaches ~40% within 12 h of 5-FU treatment (Fig. 6, Supplementary Fig. 5e). To evaluate the role of the lncRNAs under investigation in this cellular mechanism, we quantified the number of apoptotic cells following depletion of *PR-lncRNA-1* or *PR-lncRNA-10* under DNA-damage conditions. Consistent with the effect observed in proliferation, we found a significant decrease in the number of apoptotic cells measured by annexin V detection, reaching close to 50% reduction under the best knockdown conditions (Figs 6c and 4a; Supplementary Fig. 7f,g). This effect was more pronounced when *PR-lncRNA-1* was inhibited compared with *PR-lncRNA-10* inhibition (55 and 43% of reduction, respectively), and was confirmed by quantifying caspase3/7 levels under similar experimental conditions (Fig. 6d,e). We therefore concluded that *PR-lncRNA-1* and *PR-lncRNA-10* contribute to apoptosis induction by DNA damage.

p53 activity as a tumour suppressor involves also a tight control of cell cycle progression, p53 being able to control both G1 and G2/M checkpoints^31^. To further characterize the biological role of the two p53-regulated lncRNAs, we carried out cell cycle analysis of HCT116 cells depleted of the lncRNAs both in the presence or absence of DNA damage (doxo or 5-FU). When either *PR-lncRNA-1* or *PR-lncRNA-10* was depleted, and in all the experimental conditions tested, we observed a significant increase of cells in S-phase of cell cycle consistent with the increase in cell proliferation observed under the same conditions (Fig. 6f–i; Supplementary Fig. 7h,i). As for the cell proliferation assays, the differences observed in the cell cycle phase distribution between lncRNAs-depleted cells and controls were more pronounced following DNA damage compared with untreated cells (Fig. 6f–i; Supplementary Fig. 7h,i). These results suggest that both *PR-lncRNA-1* and *PR-lncRNA-10* contribute to cell cycle regulation, playing a role even when expressed at basal levels, as observed with cells not treated with DNA-damage drugs. However, their roles in cell cycle progression appeared more pronounced under DNA-damage conditions, suggesting a major role for the lncRNAs in the DNA-damage response.

Altogether, we show that the p53-regulated *PR-lncRNA-1* and *PR-lncRNA-10* contribute to the biological outcome of the p53 pathway activation by promoting apoptosis and cell cycle arrest.

### *PR-lncRNAs* constitute a tumour suppressor signature

p53 malfunction is well known to play a major role in the development of cancer. A total of 60% of non-hypermutated tumours harbour mutations in the p53 gene^32^,^33^, and 70% of colorectal carcinomas show loss of heterozygosity in 17p, where p53 gene locus resides^34^,^35^.

The results obtained for *PR-lncRNA-1* and *PR-lncRNA-10* suggest their involvement in the p53 tumour suppression function in colorectal cancer cells. We therefore hypothesized that their expression levels, as well as the expression of other p53-regulated lncRNAs, could be altered in human primary colorectal cancer specimens. To corroborate this hypothesis, we analysed the expression of the p53 directly regulated lncRNAs identified in this study in a cohort of human colon adenocarcinoma healthy tissue-paired samples.

**Figure 7:**
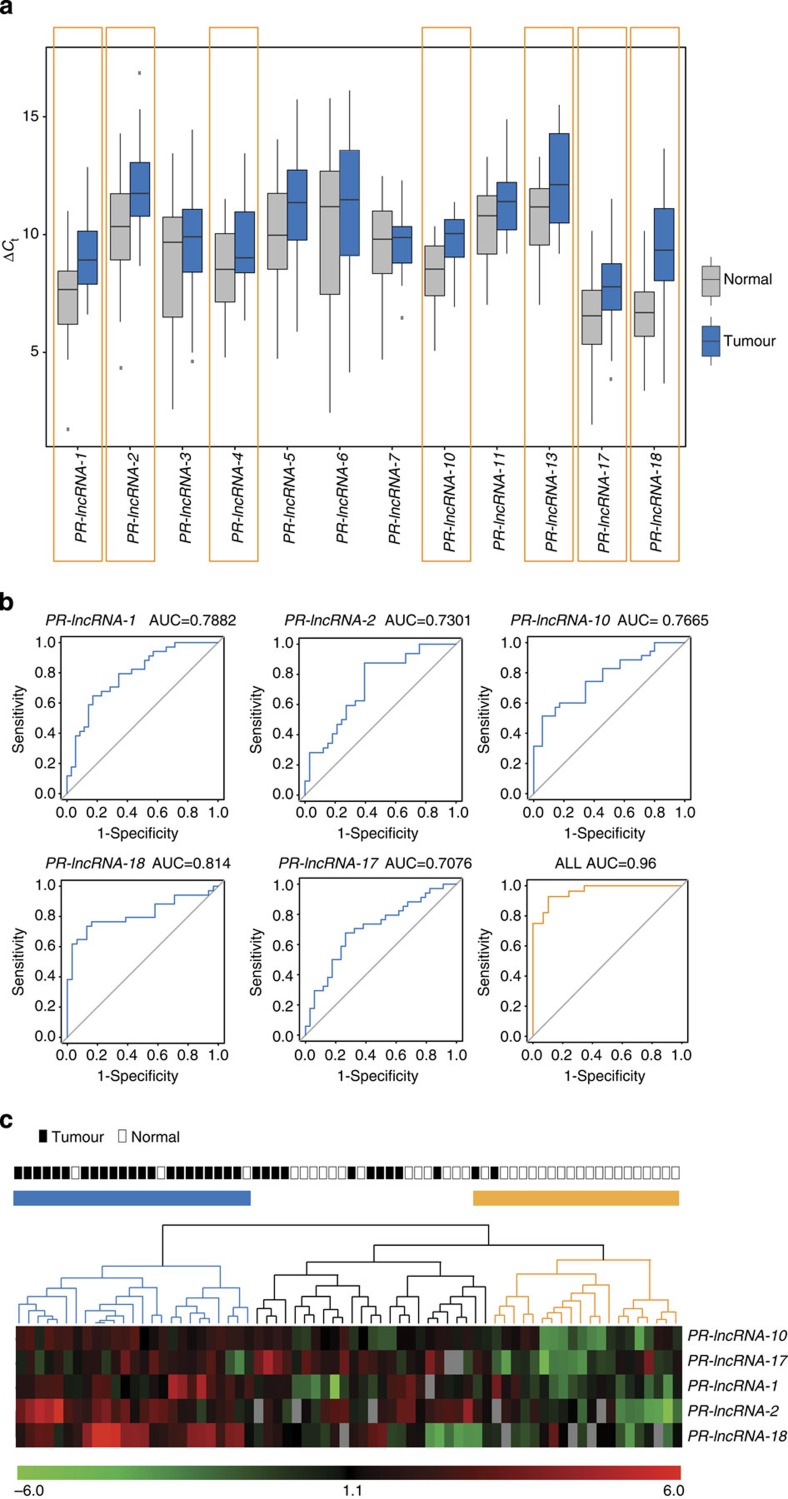
p53-regulated lncRNAs constitute a tumour-suppressor signature in colorectal cancer. (**a**) Relative expression level of p53-regulated lncRNAs in a cohort of 35 tumour samples compared with the normal peripheral tissue. The highlighted candidates are significantly downregulated in the tumours (*P*<0.01). (**b**) ROC analysis of the sensitivity and specificity for the training cohort for the seven significant p53-regulated lncRNAs. Multivariate ROC analysis for the five p53-regulated lncRNAs with AUC values >0.7. (**c**) Clustering of the expression levels of the p53-regulated lncRNAs with AUC values >0.7. Rows represent lncRNAs and columns represent patients.

In agreement with a potential role of the lncRNAs in tumour suppression, the expression levels of all the lncRNAs analysed were lower in colorectal tumours compared with normal tissue, although changes only reached significance (*P*<0.01) for 7 out of 12 lncRNAs (Fig. 7a).

To confirm that the low expression levels of the p53-regulated lncRNAs are a distinct feature of the tumour cells, we quantified the sensitivity and specificity of the lncRNAs as biomarkers by performing receiver operator characteristic (ROC) analysis (see Methods). The expression levels of five p53-regulated lncRNAs (*PR-lncRNA-1*, *PR-lncRNA-2*, *PR-lncRNA-10*, *PR-lncRNA-17* and *PR-lncRNA-18*) showed a statistically significant classificatory value (area under the curve (AUC)<0.7) that distinguished tumour from normal tissue (Fig. 7b).

Next, to improve the classification performance of the 5-lncRNA-signature, we applied a machine-learning algorithm based on logistic regression (see Methods). The higher classificatory power of the lncRNA signature was confirmed by an AUC value of 0.921 for the tenfold analysis, with a *P* value=1.9e–12 for a logistic regression model (Fig. 7b,c). These results suggest that the expression levels of this p53-regulated lncRNAs could have a diagnostic value in colorectal cancer patients.

## Discussion

Since the realization of p53’s pivotal role as a tumour suppressor, a large body of literature has identified p53-regulated genes. However, the p53 network has mostly been assembled one gene at a time and mainly focusing on genes that encode for proteins.

As a closer attempt to assess the activity of p53 on noncoding regions, a recent study has utilized p53 ectopic overexpression to relate the activity of p53 family members to the regulation of a limited number of lncRNAs^36^. However, genome-wide studies linking the activity of endogenous p53 to the expression of lncRNAs from annotated as well as non-annotated regions of the genome are still missing. Our study provides, for the first time to our knowledge, a global overview of the relationship between p53 activity and expression of lncRNAs in a human cancer cell line in response to DNA-damage treatment. We show that DNA damage inflicted by 5-FU affects the expression of hundreds of lncRNAs, a proportion similar to that found for protein-coding genes, including a large number of transcripts not previously annotated by Encode. While many of these unassigned transcripts may be artefacts of the RNA-seq assembly, others are shown to be novel lncRNAs expressed under our experimental conditions. Multiple lines of evidence support this idea: (i) some of the unassigned transcripts are annotated in other databases different from Encode^5^, (ii) their TSS is marked by histone modifications associated with active chromatin, (iii) the expression pattern and p53 regulation of several of them has been independently validated and (iv) their expression is suppressed in tumour samples compared with normal tissue.

A small proportion of the lncRNAs altered by DNA damage were found directly bound by p53 (~3%), suggesting that the rest of differentially expressed lncRNAs are likely secondary targets of p53 activation or lncRNAs regulated by DNA damage in a p53-independent manner. However, this could be a conservative estimate, as we only considered p53-biding sites that are localized within less than 10 kb of distance to the TSS, which can be more unequivocally assigned to a gene. This approach defined a high-confidence set of 18 lncRNAs that are bona fide p53 transcriptional targets.

A number of studies have shown that lncRNAs present lower conservation than protein-coding genes across species^5^,^6^. In our study, we looked for conservation between human and mouse in terms of p53 regulation and found that only 3 of the 18 p53-regulated human lncRNAs appeared to be similarly regulated in murine embryonic fibroblasts. Although this low number could be due to the differences between cell types and experimental conditions, it remains possible that some of the human lncRNAs could account for the known divergences between the murine and human p53 responses^37^. It has been proposed in fact that the more rapidly evolving lncRNA sequences may account for acquired novel biological traits^38^. On the other hand, those lncRNAs that are conserved could represent essential elements of the mammalian p53 response to DNA damage.

As representative of the p53-regulated lncRNAs, we selected *PR-lncRNA-1* and *PR-lncRNA-10* for further analyses. We found similarities and differences between both lncRNAs. *PR-lncRNA-1* and *PR-lncRNA-10* are mainly localized in the nucleus of the cells. While the regulation of *PR-lncRNA-1* by p53 appears to be conserved in mouse, *PR-lncRNA-10* is a primate-specific lncRNA. Each lncRNA regulates the expression of a different, partially overlapping, set of genes of the p53 network, contributing to the apoptotic response caused by DNA damage. Our data suggest that *PR-lncRNA-10* and, to a lesser extent, *PR-lncRNA-1* modulate the transcriptional activity of p53 by affecting its ability to recognize some of its binding sites. Although we cannot exclude other possibilities, we speculate that these nuclear PR-lncRNAs may affect the chromatin conformation at these loci, rendering them more accessible to p53 binding. This represents a novel positive-feedback mechanism in which lncRNAs induced by p53 fine-tune p53 transcriptional activity under DNA-damage conditions. However, the precise molecular mechanisms by which *PR-lncRNA-1* and *PR-lncRNA-10* contribute to enhance p53 binding to these gene loci remain to be defined.

In agreement with the results obtained with the HCT116 cell line suggesting a potential role of the lncRNAs as part of the p53 tumour suppressor response, the expression levels of seven p53-regulated lncRNAs (including *PR-lncRNA-1* and *PR-lncRNA-10*) were found significantly decreased in a cohort of colorectal cancer patient samples. This is in part an expected result since p53 gene presents alterations in the vast majority of colorectal tumours^39^. However, this observation confirms our results concerning the role played by the lncRNAs identified in this study in the p53-dependent tumour suppressor response and supports their clinical relevance.

Importantly, the regulation by p53 of these lncRNAs is not exclusive of the human cell line used in this study. We found that, similarly to that observed in HCT116 cells, the expression of the p53-regulated lncRNAs is dependent on p53 and DNA damage in other human cells analysed, including A549 human lung cancer cells (Supplementary Fig. 8a,b) and normal cells (IMR90 and BJ human fibroblasts). This observation, together with the decreased expression in colorectal tumours, leads us to conclude that the lncRNAs identified in this study are involved in the human p53 response independently of the tissue and represent a genuine subset of p53 target genes likely implicated in the tumour formation of all cancer types where p53 is affected.

## Methods

### Cell lines and DNA-damage treatment

The colon carcinoma cell lines HCT116 p53^+/+^ and p53^−/−^ were kindly provided by Dr Vogelstein’s laboratory from Johns Hopkins University, Baltimore (USA).

To obtain p53^+/+^ and p53^−/−^ MEFs, p53^LSL/LSL^ MEFs were isolated from embryos of the p53^LSL/LSL^ mouse strain and infected with AdenoCre or AdenoGFP virus for 12 h (University of Iowa) at a multiplicity of infection 5.

For DNA damage, cells were treated with 350 μM 5-FU (F6627; Sigma) or 500 nM doxorubicin hydrochloride (D1515; Sigma) for the indicated times.

### Western blot

The following antibodies were used for western blot: anti-p53 AB(DO-1) sc-126, dilution 1:1,000; anti-pP53 (S15) Cell Signaling Ref:11/2012, dilution 1:1,000; and anti-GAPDH mAbcam 9484, dilution 1:2,000. Uncropped western blot images are shown in Supplementary Fig. 9.

### RNA-seq analysis

The RNA-seq libraries were prepared from purified poly-A^+^ RNA from untreated and 5-FU-treated p53^+/+^ HCT116 cells for 4 and 12 h, including two independent samples for 12 h. Paired-end and strand-specific RNA-seq libraries were prepared according to Illumina instructions and sequenced on HiSeq 2000 (Illumina) with sequence length of 150 bp. Raw sequencing data were aligned to the human genome (hg19) using Tophat mapper, and transcripts were reconstructed using Cufflinks 2.02 (ref. 27). Annotation of the gene loci was performed using Gencode v19 as reference. Differential expression of the transcripts in response to treatment was assessed using Cuffdiff2 (ref. 23).

### p53 chromatin immunoprecipitation

A total of 10^7^ HCT116 p53^+/+^ cells were untreated or treated with 5-FU for 12 h and crosslinked with formaldehyde (10 min 1%). Glycine was then added for 5 min to a final concentration of 0.125 M, cells scrapped and collected. The cell pellet was resuspended in lysis buffer (5 mM Tris-HCl, pH 8.0, 85 mM KCl, 0.5% NP40 supplemented with protease inhibitors cocktail from Roche) and the nuclear pellet was recovered by centrifugation and resuspended in RIPA buffer (20 mM Tris-HCl, pH 7.5, 100 mM NaCl, 1 mM EDTA, 0.5% NP40, 0.5% Na-deoxycholate, 0.1% SDS supplemented with protease inhibitors cocktail). Nuclear extracts were sonicated using Bioruptor U-200.

The chromatin was precleared by adding dynabeads for 30 min at 4 °C and incubated overnight with p53 polyclonal antibody FL393 (Santa Cruz SC-6243). A total of 3 μg of dynabeads was added for 1 h at 4 °C. The antibody was coupled with dynabeads followed by four washes with LiCl wash buffer (100 mM Tris, pH 7.5, 500 mM LiCl, 1% NP40, 1% Na-deoxycholate) and 1 × with TE (10 mM Tris-HCl, pH 7.5, 1 mM EDTA, pH 8.0). DNA was eluted for 1 h at 37 °C in Elution Buffer (1% SDS, 0.1 M NaHCO_3_), reverse X linked, purified and analysed by qPCR or used for sequencing library preparation.

### p53 motif analysis

Genes were differentially selected for the *de novo* motif search performed with MEME-ChIP^40^ using the binding site sequences corresponding to these enriched regions with default parameters. TOMTOM tool of MEME-ChIP was used for the comparison of obtained DNA motifs against JASPAR database of known motifs.

### Microarray analysis

For gene expression profiling, total RNA was extracted and hybridized to Affymetrix Human Transcriptome Array 2.0. Background correction and normalization were done using RMA (Robust Multichip Average) algorithm^41^ using Affymetrix Power Tools. After quality assessment, a filtering process was performed to eliminate low-expression probe sets. Applying the criterion of an expression value greater than 16 in two samples for each experimental condition, 41,697 probe sets were selected for statistical analysis. R and Bioconductor were used for preprocessing and statistical analysis. LIMMA (Linear Models for Microarray Data)^42^ was used to find out the probe sets that showed significant differential expression between experimental conditions. Genes were selected as significant using a B-statistic cut-off *B*>0.

Functional enrichment analysis of Gene Ontology (GO) categories was carried out using standard hypergeometric test^43^. The biological knowledge extraction was complemented through the use of Ingenuity Pathway Analysis (Ingenuity Systems; www.ingenuity.com).

### qPCR primers and ASOs

The qPCR primers and ASO sequences used in this study are listed in Supplementary Table 9.

All ASOs were designed and provided by ISIS Pharmaceuticals. All were 20 nt in length and chemically modified with phosphorothioate in the backbone, five 2′-O-methoxyethyl residues at each terminus and a central deoxynucleotide region of 10 residues (5-10-5 gapmer). ASOs were synthesized using an Applied Biosystems 380B automated DNA synthesizer (PerkinElmer Life and Analytical Sciences-Applied Biosystems).

### Cell proliferation assays

For proliferation analysis, 1,000 cells were plated per well in 96-well plates and assessed with a CellTiter96 Aqueous Non-Radioactive Cell Proliferation Assay (MTS) Kit G3581 (Promega).

### Apoptosis and cell cycle analysis

At 24 h after transfection, 1 × 10^5^ cells were plated in 96-well white microplates and treated for 24 h with 385 μM 5-FU. Apoptosis was determined by quantification with caspase-Glo 3/7 reagent (Promega) using a FLUOstar Optima luminometer, and with annexin V fluorescence-activated cell sorting using an Apoptosis Detection Kit I (cat-559763; BD Biosciences).

For cell cycle analysis, cells were labelled with propidium iodide (PI) and measured in the FACSCalibur flow cytometer (BD Biosciences). Data represent the mean±s.d. of a minimum of three biological replicates.

### Human samples

Human colorectal carcinoma samples were provided by the Basque Biobank for Research-OEHUN (http://www.biobancovasco.org). Samples were processed following standard operation procedures with appropriate ethical approval by the Ethics Committee for Clinical Research of Donostia Hospital. An informed consent was obtained from all subjects. RNA was extracted with RNEASY MINI KIT (Qiagen) and reverse transcription was performed using random priming and Superscript Reverse Transcriptase (Life Technologies), according to the manufacturer’s guidelines.

The ▵*C*_t_ value was determined by subtracting the *GAPDH C*_t_ value from the p53-regulated lncRNAs’ *C*_t_ value.

The performance as biomarker of each lncRNA was evaluated using ROC analysis^44^.

An algorithm based on logistic regression was applied to the obtained 5-lncRNA-signature classification^45^.

### Subcellular fractionation

A total of 10^7^ cells were trypsinized and washed once with cold PBS, aliquoted in two tubes and collected by centrifugation at 1,000*g* for 5 min at 4 °C. One cell pellet represented the whole-cell extract, while the other one was processed for the remaining subcellular fractions. Both pellets were resuspended in 500 μl of Buffer A (10 mM Tris-HCl, pH 7.5, 1.5 mM MgCl_2_, 140 mM NaCl, 0.05 IGEPAL supplemented with protease inhibitor cocktail and SuperaseIN 10 U ml^−1^), incubated for 10 min on ice and kept for subsequent RNA extraction. A total of 500 μl of Buffer A plus sucrose (10 mM Tris-HCl, pH 7.5, 1.5 mM MgCl2, 140 mM NaCl 0.05% IGEPAL, 50% Sucrose) was added to the bottom of a clean eppendorf tube and the upper phase (whole-cell extract resuspended in Buffer A) was gently added to this tube preventing the mix of the two phases and centrifuged for 10 min at 4 °C and 12,000*g* to obtain nuclear and cytoplasmic fractions. Around 500 μl of the upper phase (cytoplasmic fraction) was collected and the rest was discarded, leaving the pellet (nuclear fraction). Total nuclear fraction was resuspended in 500 μl of Buffer B (10 mM Tris, 100 mM NaCl, 1 mM EGTA, 300 mM sucrose, 0.5 mM NaVO_3_, 50 mM NaF, 1 mM phenylmethylsulphonyl fluoride, 0.5% triton X-100, protease inhibitor cocktail and SuperasIN) and incubated for 10 min on ice to permeabilize the cells. To separate nuclear soluble from nuclear insoluble fraction, sample was centrifuged at 2,000 *g* for 5 min at 4 °C and the supernantant (nuclear *s*oluble fraction) and pellet (nuclear insoluble/chromatin fraction) were collected. The nuclear insoluble fraction was resuspended in Buffer A and finally 1 ml of Trizol was added to all tubes for subsequent RNA extraction.

### RNA fluorescence *in situ* hybridization

RNA fluorescence *in situ* hybridization for *PR-lncRNA-1* and *PR-lncRNA-10* detection was performed using a pool of 48 fluorescent probes purchased from Stellaris Biosearch Technologies, following the manufacturer’s instructions.

### Statistical analysis

Experimental data are represented as the mean±s.d. of a minimum of three biological replicates and were compared using Student’s *t*-test. Significant *P* values are indicated with asterisks as follows: *P<0.05, **P<0.01 and ***P<0.001.

## Author contributions

M.H. and Y.S. designed the research; Y.S., F.P.M., O.M.-B. and J.G. performed the research; V.S. and A.A. performed the data analysis; A.M. and L.B. contributed to the collection of patient samples; S.G. contributed with design, testing and production of reagents; and M.H. and Y.S. wrote the paper.

## Additional information

**How to cite this article**: Sánchez, Y. *et al*. Genome-wide analysis of the human p53 transcriptional network unveils a lncRNA tumour suppressor signature. *Nat. Commun.* 5:5812 doi: 10.1038/ncomms6812 (2014).

**Accession codes:** Primary data from RNA-seq, p53 ChIP-seq and microarray analyses have been deposited in the Gene Expression Omnibus under the accession code GSE58528.

## Supplementary Material

Supplementary Information Supplementary Figures 1-8, Supplementary Methods and Supplementary References

Supplementary Data 1 Transcripts differentially expressed upon DNA damage treatment with 5-FU identified by the RNA-seq analysis.

Supplementary Data 2 Genes bound by p53 at 0h or 12 h of DNA damage treatment determined by p53 ChIP-seq analysis.

Supplementary Data 3 Protein-coding genes with at least one transcript differentially expressed and bound by p53.

Supplementary Data 4 LncRNAs differentially expressed by DNA damage treatment and bound by p53.

Supplementary Data 5 Genes bound by p53 in MEFs.determined by p53 ChIP-seq.

Supplementary Data 6 Genes differentially expressed bound by p53 in human and murine cells.

Supplementary Data 7 Genes affected by PR-lncRNA-1 and PR-lncRNA-10 depletion by ASO transfection of HCT116 p53 + / + treated with 5FU for 12h (B>0).

Supplementary Data 8 Genes differentially expressed upon DNA damage (RNA-seq analysis) and affected by PR-lncRNA-1 or PR-lncRNA-10 inhibition.

Supplementary Data 9 Sequences of oligonucleotides used in this study .
