# Supplementary material for: Genome-wide analysis of the human p53 transcriptional network unveils a lncRNA tumour suppressor signature
Source: Nat Commun. 2014 Dec 19;5:5812. doi: 10.1038/ncomms6812 (PMC4284803; doi:10.1038/ncomms6812)
Supplement: Supplementary Information — Supplementary Figures 1-8, Supplementary Methods and Supplementary References [file ncomms6812-s1.pdf]

SUPPLEMENTARY INFORMATION

SUPPLEMENTARY FIGURES

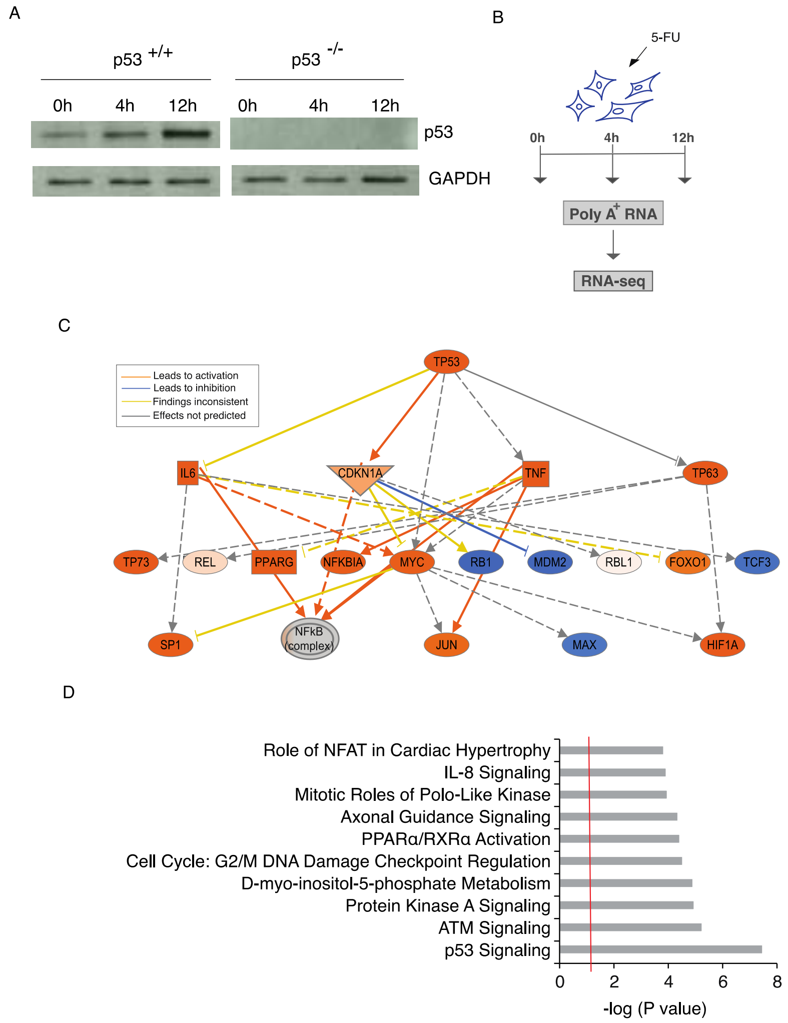

Supplementary Figure 1. Genome-wide transcriptional analysis of the DNA damage response

(A) Western blot analysis of p53 and p21 in the HCT116 p53 $^{+}/^{+}$ and p53 $^{-}/^{-}$ cell lines untreated or treated with 5FU for 4, 8 and 12h. β-actin is shown as a loading control. (B) Experimental layout to identify human polyadenylated RNAs affected by DNA damage. HCT116 cells were left untreated or treated with 5-fluorouracil (5-FU) for 4 and 12 hours to induce DNA damage. Pathways enrichment (D) and Upstream regulator prediction (C) for genes differentially expressed following 5-FU treatment based on the fold changes obtained by RNA-seq analysis. Data were analyzed with Ingenuity Pathway Analysis.

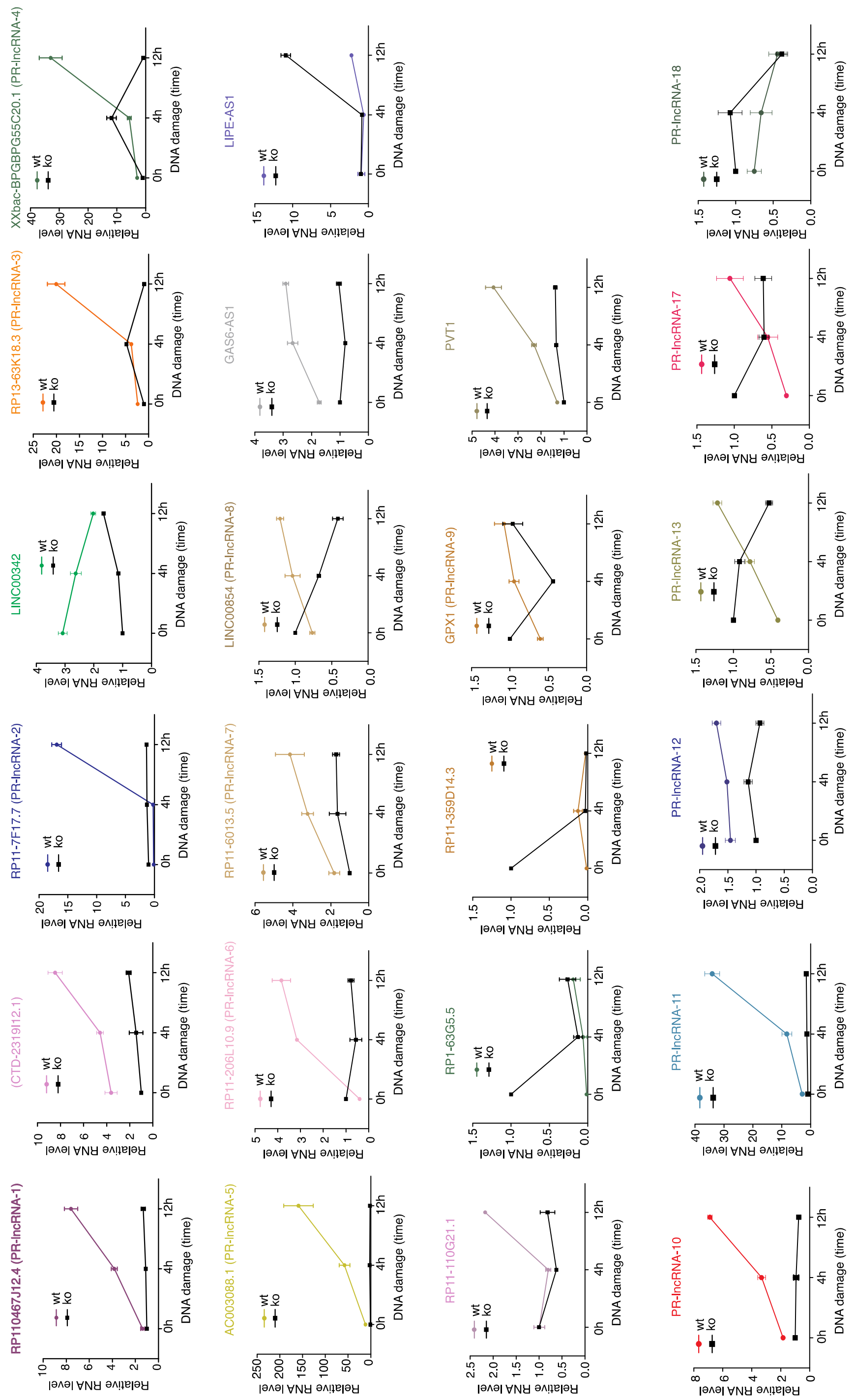

Supplementary Figure 2. p53-regulated lncRNAs in response to the DNA damage. Validations by qRT-PCR of annotated and non-annotated lncRNAs differentially expressed (p<0.01) by the DNA damage 5-FU treated for 12h in the p53 $^{+}/^{+}$ and p53 $^{-}/^{-}$ HCT116 cell line. Values are normalized to GAPDH and relative to untreated p53 $^{-}/^{-}$ cell line. Values are the average of three replicates ±SD.

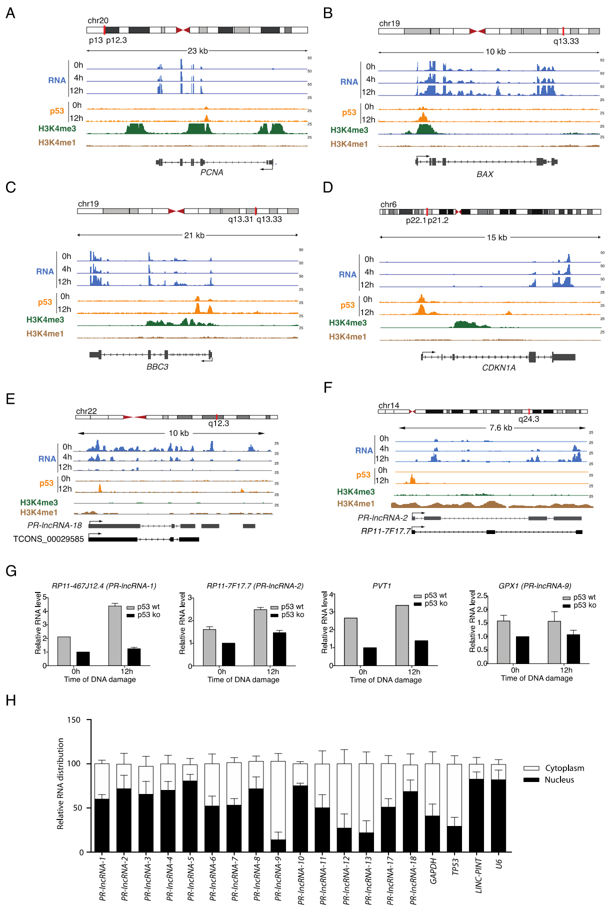

Supplementary Figure 3. p53-regulated genes upon DNA damage treatment. Schematic representation of the hromosomal location of *PCNA* (A), *BAX* (B), *BBC3* (C), *CDKN1A* (D), *PR-lncRNA-18* (E) and *PR-lncRNA-2* (F) gene loci, RNA expression peaks obtained by RNA-seq, H3K4me3 and H3K4me1 ChIP-seq peaks of HCT116 cells from ENCODE and transcript isoforms as assembled by Cufflinks 2.02. (G) Relative expression levels for *PR-lncRNA-1*, *PR-lncRNA-3*, *PR-lncRNA-8* and PVT1 in p53-restored or $p53^{-/-}$ LSL Mouse Embryonic Fibroblast (MEFs) treated with 5FU for 12h. Values are normalized by GAPDH and are the mean (±SD) of three different experiments. (H) Relative subcellular distribution of the indicated RNAs in the nuclear and cytoplasmic fractions of HCT116 $p53^{+/+}$ treated with 5FU for 12h determined by quantitative real time (RT-qPCR). Data represent the mean (±SD) of at least three independent experiments.

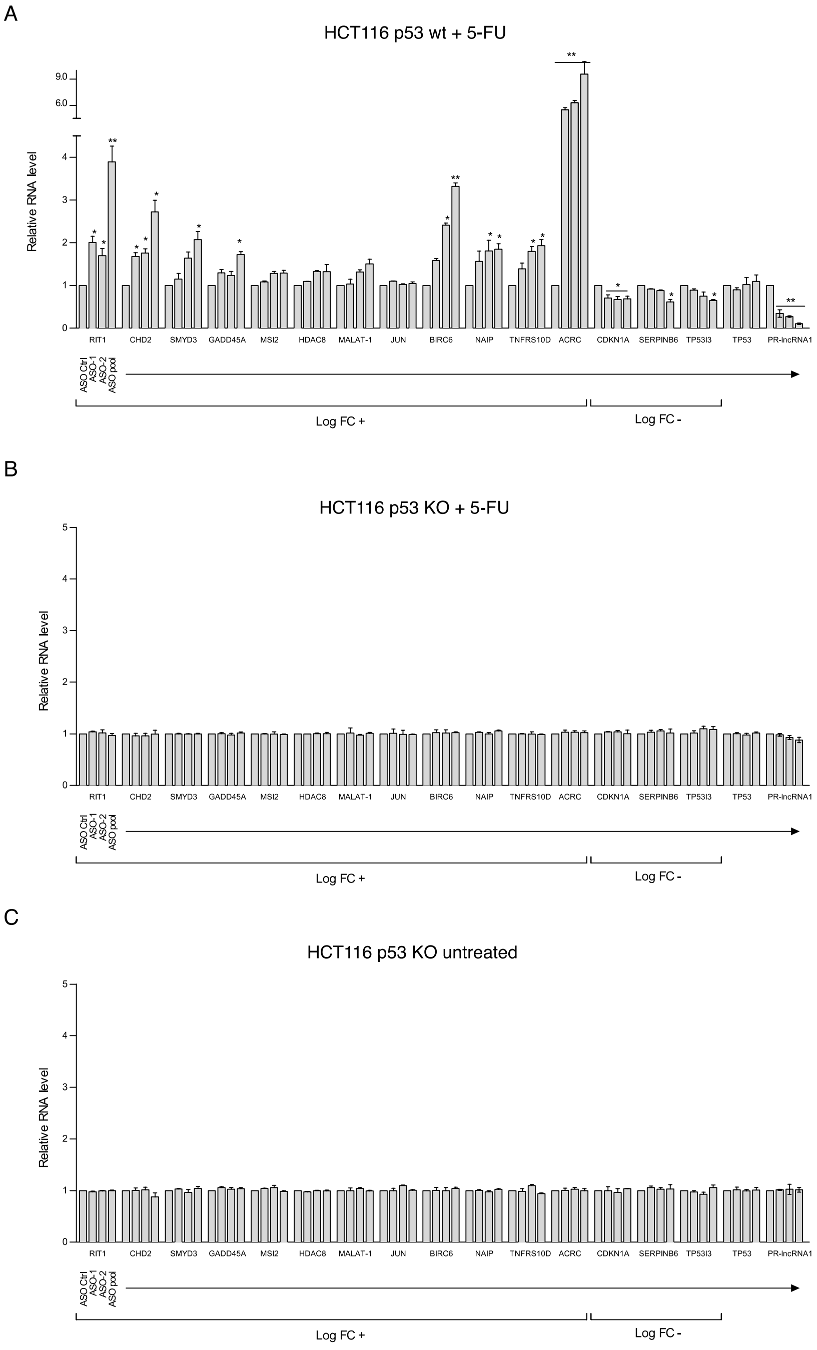

Supplementary Figure 4. ***PR-lncRNA-1* affects the expression of genes of the p53 transcriptional network**.

Validations by qRT-PCR of the expression of genes upon depletion of *PR-lncRNA-1* in HCT116 p53 $^{+}/^{+}$ treated with 5-FU (A) HCT116 p53$^{-/-}$ treated with 5-FU (B) and HCT116 p53$^{-/-}$ untreated (C). Values represent the mean (±SD) of at least three independent experiments. Significance was determined by two-tailed unpaired *t*-test. *, p<0.05; **, p<0.01; and ***, p<0.001.

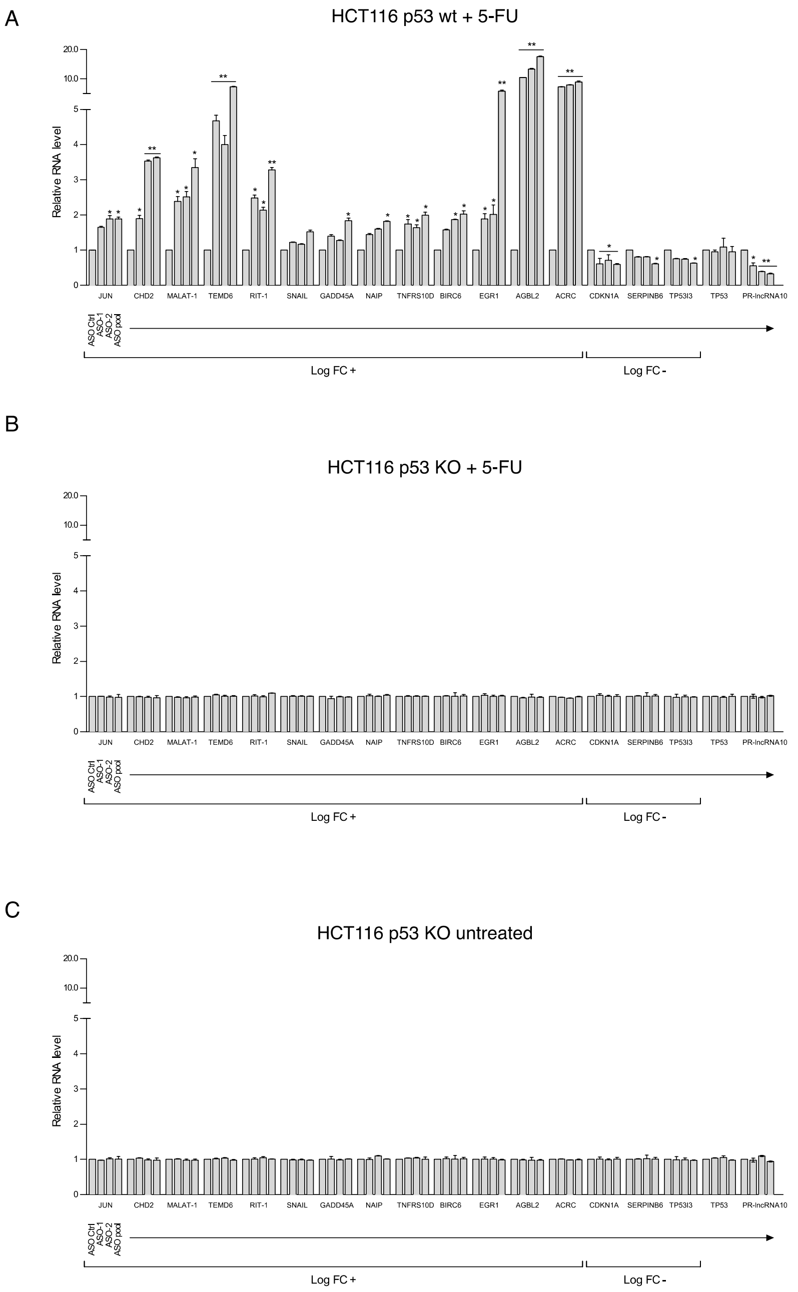

Supplementary Figure 5. ***PR-lncRNA-10* affects the expression of genes of the p53 transcriptional network**.

Validation by qRT-PCR of the genes detected as significantly affected by depletion of *PR-lncRNA-10* in HCT116 p53 $^{+}/^{+}$ treated with 5-FU (A) HCT116 p53$^{-/-}$ treated with 5-FU (B) and HCT116 p53$^{-/-}$ untreated (C). Values represent the mean (±SD) of at least three independent experiments. Significance was determined by two-tailed unpaired *t*-test. *, p<0.05; **, p<0.01; and ***, p<0.001.

A

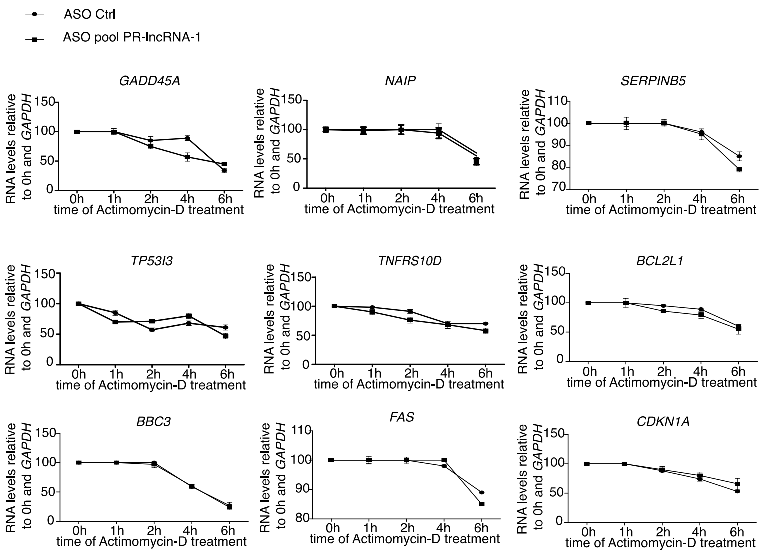

B

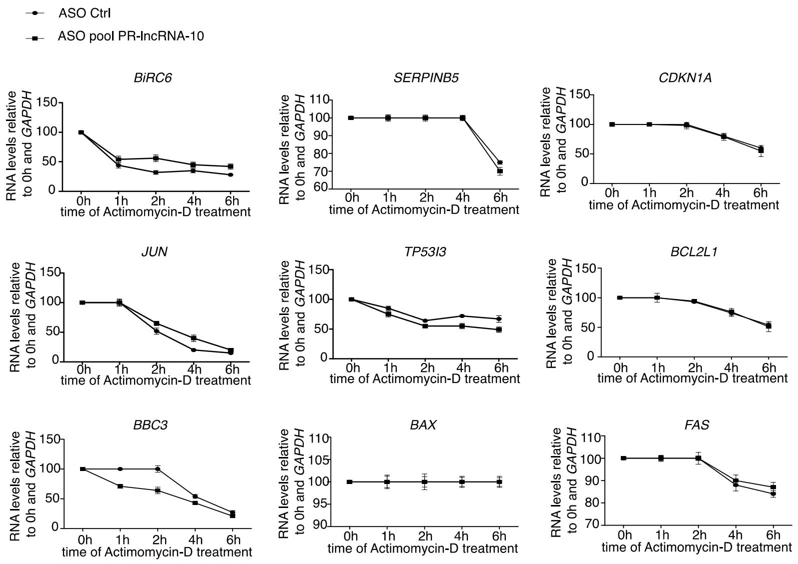

Supplementary Figure 6. mRNA Stability.

mRNA stability of the genes affected by the knockdown of *PR-lncRNA-1* or *PR-lncRNA-10*. HCT116 $p53^{+/+}$ were transfected with the ASO pool for the *PR-lncRNA-1* (A) and *PR-lncRNA-10* (B) for 24h and treated with 5-FU for 12h. Actinomycin D was added for the indicated times. mRNA levels were normalized to GAPDH mRNA and values shown as percentage of $t = 0$ h of actinomycin D treatment. Data represent the mean (±SD) of at least three independent experiments.

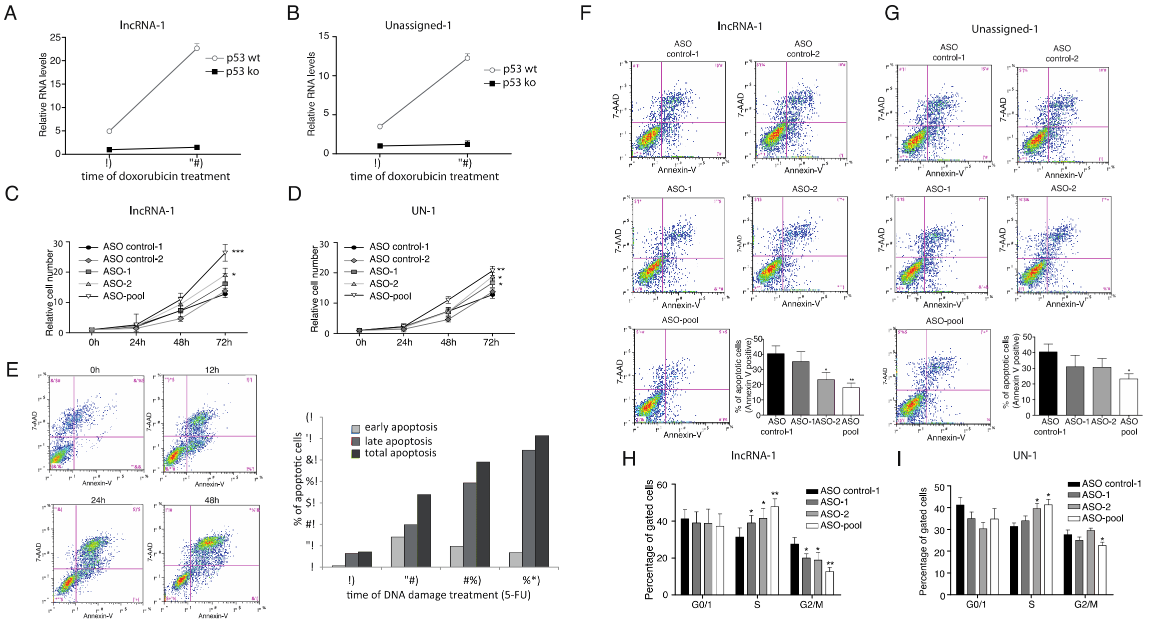

Supplementary Figure 7. *PR-lncRNA-1* and *PR-lncRNA-10* modulate cell proliferation and apoptosis.

A and B. *PR-lncRNA-1* (A) and *PR-lncRNA-10* (B) expression determined by qRT-PCR in response to 500nM Doxorubicin treatment in the p53 $^{+}/^{+}$and p53$^{-}/^{-}$ HCT116 cell lines for 12h.

C and D. Relative number of untreated HCT116 p53$^{+}/^{+}$ cells transfected with the indicated ASOs. Cell proliferation was measured by MTS assay up to 72 hours. Values are normalized to day 0 and are the mean of three different experiments +/- SD

E. Quantification of apoptotic cells by FACS detection of Annexin V staining of HCT116 cells after treatment with 5-FU for the indicated times.

F and G. Percentage of apoptotic cells treated with the indicated ASOs for *PR-lncRNA-1* (F) or *PR-lncRNA-10* (G) depletion and treated with 350uM 5-FU for 12h.

H and I. Cell cycle phases distribution analyzed by propidium iodide staining in the untreated HCT116 p53$^{+}/^{+}$ cells with the transfection of the indicated ASOs for *PR-lncRNA-1* (H) or *PR-lncRNA-10* (I) depletion. All graphs represent the mean ±SD of at least three independent experiments. Significance was determined by two-tailed unpaired *t*-test. *, p<0.05; **, p<0.01; and ***, p<0.001.

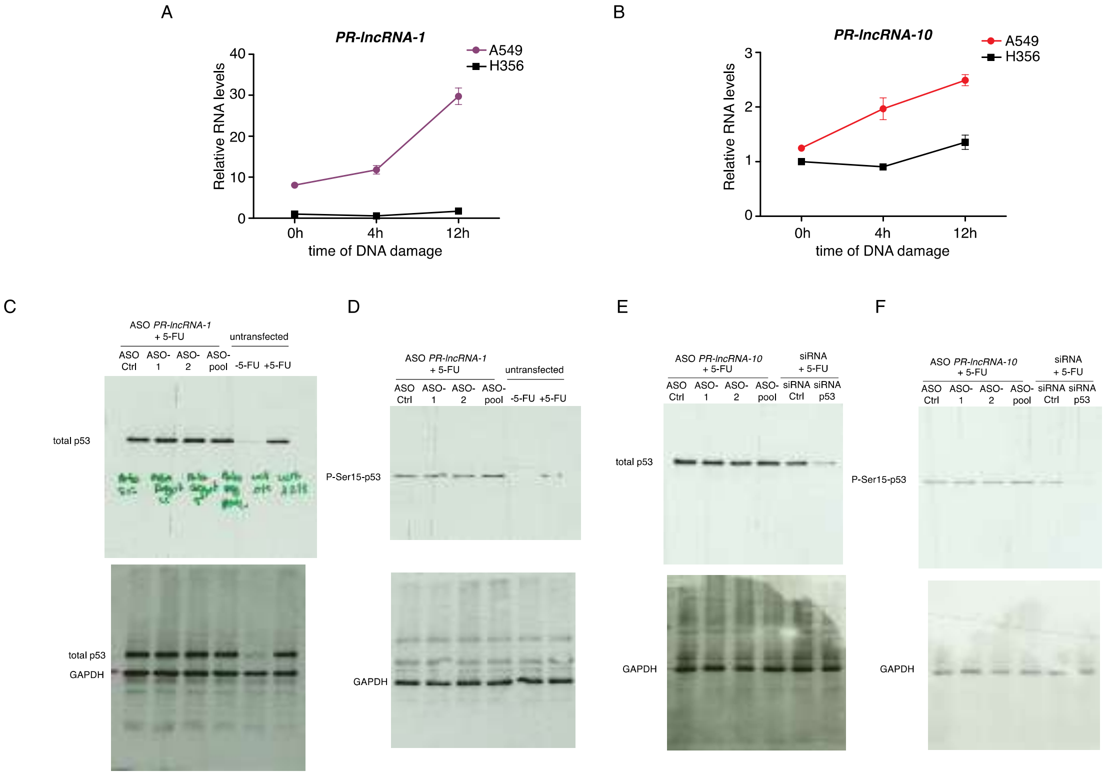

Supplementary Figure 8.

A and B. Expression of *PR-lncRNA-1* (A) and *PR-lncRNA-10* (B) in A549 (p53 wt) and H358 (p53 ko) lung cancer cell lines detected by qRT-PCR Values are normalized to *GAPDH* and are the average of three replicates ±SD.

C-F. Western blot analysis of total p53 and phospho-p53 (Serine 15) on HCT116 cells transfected with ASOs for *PR-lncRNA-1* (C-D) or *PR-lncRNA-10* (E-F) depletion, and treated with the 5FU for 12h as indicated. GAPDH is shown as a loading control.

SUPPLEMENTARY MATERIALS AND METHODS

RNA-seq data analysis

Raw sequencing data generated on the Illumina genome Analyzer II were processed using the following workflow: (1) the quality of the samples was verify using FastQC software; (2) the preprocessing of reads included elimination of contaminant adapter

substrings with Scythe and quality-based trimming using Sickle; (3) the alignment of reads to the human genome (hg19) was performed using Tophat2 mapper [1] (4) the transcript assembly and quantification using FPKM of genes and transcripts was carried out with Cufflinks2 [2]; (5) the annotation of the gene locus obtained was performed using Cuffmerge with Gencode v19 as reference; (6) differential expression analysis was performed using Cuffdiff2 and selected the transcript with a $p<0.01$ between untreated and treated p53 HCT116 cells for 12h [2]. Further analysis and graphical representations have been performed using R/Bioconductor [3].

ChIP-seq data analysis

Reads were aligned using Bowtie2 [4] to the reference genome (hg19 for human samples). Peak detection for p53 ChIP-Seq samples were performed using MACS 1.4.2 [5] with default parameters but without input (the enrichment regions are identified for WT0h and WT12h conditions using the background of each experiment as reference in the Poisson model). The annotation of the obtained peaks was done using the Bioconductor package ChIPpeakAnno [6] using as reference the annotation obtained from the combination of Gencode v19 and Cufflinks genes. Overlapping genes were discarded and only regions as far as 10kb from TSS are considered for further analyses.

Public data from mouse p53 ChIP-Seq experiments were downloaded from Gene Expression Omnibus (GEO) database (accession code GSE46240) and analyzed using previously described pipeline. In order to compare human and mouse results, peak coordinates from mm10 were transformed into hg19 coordinates using liftOver tool from UCSC, and the converted regions were annotated using ChIPpeakAnno.

Histone modification ChIP-Seq experiments of H3K27ac, H3K4me1 andH3K4me3 for HCT-116 cell line were downloaded from ENCODE project [7], with GEO accession codes GSE31755 and GSE35583.

Coverage signals used to represent heatmap density maps and centered peak regions were generated using seqMINER [8] and visualized with Genesis [9] and ggplot2 package from Bioconductor.

Microarray hybridization and data analysis

The cells were harvested with TRIzol Reagent (Invitrogen) and the RNA was extracted according to the manufacturer's instructions. As a last step of the extraction procedure, the RNA was purified with the RNeasy Mini-kit (Qiagen, Hilden, Germany). Before cDNA synthesis, RNA integrity from each sample was confirmed on Agilent RNA Nano LabChips (Agilent Technologies). The sense cDNA was prepared from 300 ng of total RNA using the Ambion® WT Expression Kit. The sense strand cDNA was then fragmented and biotinylated with the Affymetrix GeneChip® WT Terminal Labeling Kit (PN 900671). Labeled sense cDNA was hybridized to the Affymetrix Human Transcriptome Array 2.0 microarray according to the manufacturer protocols and using GeneChip® Hybridization, Wash and Stain Kit. Genechips were scanned with the Affymetrix GeneChip® Scanner 3000.

Both background correction and normalization were done using RMA (Robust Multichip Average) algorithm [10] using Affymetrix Power Tools (APT). After quality assessment a filtering process was performed to eliminate low expression probe sets. Applying the criterion of an expression value greater than 16 in 2 samples for each experimental condition, 41697 probe sets were selected for statistical analysis. R and Bioconductor were used for preprocessing and statistical analysis. LIMMA (Linear

Models for Microarray Data) [11] was used to find out the probe sets that showed significant differential expression between experimental conditions. Genes were selected as significant using a B statistic cut off B>1.

Functional enrichment analysis of Gene Ontology (GO) categories was carried out using standard hypergeometric test [12]. The biological knowledge extraction was complemented through the use of Ingenuity Pathway Analysis (Ingenuity Systems, www.ingenuity.com), which database includes manually curated and fully traceable data derived from literature sources

Measurement of mRNA stability

mRNA stability was measured by actinomycin D chases. Actinomycin D (5 ug/ml) was added to cells for the indicated time, RNA was extracted and analyzed by qRT-PCR.

Oligonucleotides

Sequences of antisense oligos (ASOs) and PCR primers are shown in Supplementary Table 9.

Supplementary References:

1. Kim, D. *et al*. TopHat2: accurate alignment of transcriptomes in the presence of insertions, deletions and gene fusions. *Genome Biol.* **14**, R36 (2013).

2. Trapnell, C. *et al*. Transcript assembly and quantification by RNA-Seq reveals unannotated transcripts and isoform switching during cell differentiation. *Nat. Biotechnol.* **28**, 511-515 (2010).

3. Gentleman, V. Carey S Dudoit R, Irizarry and Huber. in 1-473, 2005).

4. Langmead, B. & Salzberg, S. L. Fast gapped-read alignment with Bowtie 2. *Nat. Methods* **9**, 357-359 (2012).

5. Zhang, Y. *et al*. Model-based analysis of ChIP-Seq (MACS). *Genome Biol.* **9**, R137-2008-9-9-r137. Epub 2008 Sep 17 (2008).

6. Zhu, L. J. *et al*. ChIPpeakAnno: a Bioconductor package to annotate ChIP-seq and ChIP-chip data. *BMC Bioinformatics* **11**, 237-2105-11-237 (2010).

7. ENCODE Project Consortium *et al*. An integrated encyclopedia of DNA elements in the human genome. *Nature* **489**, 57-74 (2012).

8. Ye, T. *et al*. seqMINER: an integrated ChIP-seq data interpretation platform. *Nucleic Acids Res.* **39**, e35 (2011).

9. Sturn, A., Quackenbush, J. & Trajanoski, Z. Genesis: cluster analysis of microarray data. *Bioinformatics* **18**, 207-208 (2002).

10. Irizarry, R. A. *et al*. Summaries of Affymetrix GeneChip probe level data. *Nucleic Acids Res.* **31**, e15 (2003).

11. Smyth, G. K. Linear models and empirical bayes methods for assessing differential expression in microarray experiments. *Stat. Appl. Genet. Mol. Biol.* **3**, Article3 (2004).

12. Draghici S. in (ed Chapman Hall /CRC, l.), 2003).
